# Oxidative Balance Scores (OBSs) Integrating Nutrient, Food and Lifestyle Dimensions: Development of the NutrientL-OBS and FoodL-OBS

**DOI:** 10.3390/antiox11020300

**Published:** 2022-01-31

**Authors:** Ángela Hernández-Ruiz, Belén García-Villanova, Eduardo J. Guerra-Hernández, Cayetano Javier Carrión-García, Pilar Amiano, María-José Sánchez, Esther Molina-Montes

**Affiliations:** 1 Department of Nutrition and Food Science, Faculty of Pharmacy, Campus Universitario de Cartuja S/N, University of Granada, 18071 Granada, Spain; ahernandez@finut.org (Á.H.-R.); ejguerra@ugr.es (E.J.G.-H.); cjaviercargar@yahoo.es (C.J.C.-G.); memolina@ugr.es (E.M.-M.); 2 Nutrition and Food Science Doctorate Program (RD 99/2011), University of Granada, 18071 Granada, Spain; 3 Iberoamerican Nutrition Foundation (FINUT), Armilla, 18016 Granada, Spain; 4 Ministry of Health of the Basque Government, Sub Directorate for Public Health and Addictions of Gipuzkoa, 20014 San Sebastian, Spain; epicss-san@euskadi.eus; 5 Epidemiology of Chronic and Communicable Diseases Group, Biodonostia Health Research Institute, 20014 San Sebastian, Spain; 6 CIBER of Epidemiology and Public Health (CIBERESP), 28029 Madrid, Spain; mariajose.sanchez.easp@juntadeandalucia.es; 7 Instituto de Investigación Biosanitaria ibs.GRANADA, 18012 Granada, Spain; 8 Andalusian School of Public Health (EASP), 28029 Granada, Spain; 9 Department of Preventive Medicine and Public Health, University of Granada, 18071 Granada, Spain; 10 Biomedical Research Centre, Institute of Nutrition and Food Technology (INYTA) ‘José Mataix’, University of Granada, 18071 Granada, Spain

**Keywords:** antioxidant, oxidative stress, Mediterranean diet, biomarker, diet quality

## Abstract

Oxidative Balance Scores (OBS) are tools that allow us to assess the individual’s antioxidant state by ranking both antioxidant and pro-oxidant components of dietary and lifestyle factors. Our aim was to develop novel OBSs accounting for either the global supply of nutrient antioxidants in the diet, or the intake of antioxidant-rich foods, in combination with lifestyle factors. Pro-oxidant factors were also considered. Within two centers of the Spanish European Prospective Investigation into Cancer and Nutrition (EPIC) study, EPIC-Granada and EPIC-Gipuzkoa (N = 14,756 participants), we developed the Nurient, Food and Lifestyle OBS (NutrientL-OBS and FoodL-OBS), and their simplified versions (solely with dietary or lifestyle factors, the Nutrient-OBS, Food-OBS and L-OBS). Their antioxidant potential was evaluated considering their relationship with: (i) 20 scores of adherence to the Mediterranean Diet (MD); and, (ii) 25 biomarkers of antioxidant nutrients (ascorbic acid, β-carotene, etc.), inflammation (CRP, TNF-alpha, etc.) and oxidative stress (uric acid), among 210 participants. Spearman correlation and multivariate linear regression analyses were applied to analyze these associations. Some statistically significant relationships were encountered between the NutrientL-OBS and the FoodL-OBS with the MD scores, and with ascorbic acid (per one-unit increase in OBS: β = 0.012 and 0.015; *p* = 0.022 and 0.008, respectively) and CRP (per one-unit increase in both OBS: β = −0.02; *p* = 0.02); the latter appeared to be restricted to the OBS´s lifestyle components. In conclusion, the NutrientL- and FoodL-OBSs and their sub-versions are related to antioxidant-rich dietary patterns and to biomarkers of antioxidant nutrient intake and inflammation, supporting that these tools are valid to assess the individual´s oxidative/antioxidant status.

## 1. Introduction

Oxidative Stress (OS) arises from an imbalance between the production of reactive oxygen species (ROS) more commonly referred to as free radicals, and the antioxidant defense system [1]. The accumulation of free radicals can cause damage to the structure of cells and their functions by oxidative degradation of lipids, proteins, or DNA [2,3]. Therefore, these free radicals boost the development of OS-related diseases, including cancer [4]. By contrast, the antioxidant defense system decreases the pool of ROS and subsequently the risk of such diseases, as well as the aging process [5]. Moreover, there is a connection between OS and inflammation; thus, OS may drive inflammation-related diseases, too [6].

Pro-oxidant factors are capable to induce OS by the generation of ROS or by decreasing the antioxidant system defense activity. For example, xenobiotics of tobacco smoke, obesity, and some foods are potential pro-oxidant factors [7,8,9]. Excessive caloric intake, which is related to insulin resistance and obesity development, has been shown to cause oxidation in adipose tissue and is therefore also linked to OS [10]. Most of these pro-oxidant factors are modifiable. By contrast, antioxidant factors have potential to shifting the balance towards a less pro-oxidant state [11]. Some antioxidant factors to note are: dietary antioxidants, healthy weight maintenance, non-smoking, being physically active, and non-consumption of alcoholic beverages [12,13,14,15,16]. On the other hand, a dietary pattern rich in plant-based foods is essential for protection against OS. These foods provide antioxidant vitamins (C, E, and A) including carotenoids and phenolic compounds, which together account for a high total antioxidant capacity (TAC) in the diet [17]. In fact, about 90% of the dietary TAC comes from plant foods and beverages [18]. Nowadays, the assessment of the dietary TAC provides global information on the content of antioxidant compounds in the diet. More specifically, TAC accounts for the antagonistic and synergistic effect of all antioxidant compounds in the diet, and represents a greater value than the sum of its parts [19]. Therefore, dietary TAC is considered to be highest in a diet of antioxidant enriched foods [20]. It is also widely acknowledged that a high dietary TAC is, to some extent, related to increased levels of antioxidants and TAC in the body [21]. There are several assays available to measure TAC in foods and in the diet, such as the total radical-trapping antioxidant parameter (TRAP) and the ferric-reducing antioxidant power (FRAP), both of which undergo different antioxidant reactions [3,19].

Based on the above, there are both pro-oxidant and antioxidant factors, that interact together, determining the individual´s oxidative balance. Oxidative Balance Scores (OBSs) were created to globally evaluate this status. The whole idea is to gauge the complexity of the assessment of the oxidative status resulting from exposures to pro- and antioxidant factors in a one-dimensional measurement tool. These scores usually combine dietary components (antioxidant vitamins and flavonoids, as well as pro-oxidants such as iron), components related to lifestyles (antioxidants such as physical activity (PA), or pro-oxidants such as overweight/obesity) and sometimes even medication components (consumption of non-steroidal anti-inflammatories as antioxidant components, for example) [22]. The first OBS was developed by Van Hoydonck et al., which included only three components, two antioxidants (β-carotene and vitamin C) and one pro-oxidant factor (iron) [23]. Later, the OBS proposed by Goodman et al, included 9 antioxidant and 3 pro-oxidant factors [24]. More than 20 adaptations of these OBSs have been published thereafter, in an attempt to improve this assessment by selecting different components or by adopting different scoring systems. Together, these tools have been extensively applied in epidemiological studies to assess associations between oxidative status and the risk of developing several chronic diseases [22]. However, to date, there is no consensus definition of an OBS, and little has been done to prove whether these tools truly reflect the antioxidant and oxidant status.

Our aim was to develop different OBSs tools for the assessment of the individual’s oxidative balance status, considering dietary and lifestyle factors, as well as solely food components. Furthermore, we sought to validate them in terms of their relationship with antioxidant-rich dietary patterns such as the MD and biomarkers of antioxidant status and inflammation. For this purpose, we used data of biomarkers, pro-oxidant and antioxidant factors, available in the European Prospective Investigation into Cancer and Nutrition (EPIC) study, within the EPIC-Granada & EPIC-Gipuzkoa study.

## 2. Materials and Methods

### 2.1. Study Population

A cross-sectional study was conducted within two centers of the Spanish EPIC study, EPIC-Granada and EPIC-Gipuzkoa. In brief, EPIC is a large European multicentric study that was designed to identify risk factors linked to the development of cancer and other chronic diseases. EPIC-Spain is one of the participating countries, contributing with over forty-four hundred participants (aged between 32 and 69 years) to the overall study. Participants were recruited by five centers (Gipuzkoa, Navarra and Asturias in the North of Spain, and Murcia and Granada in the South of Spain) between 1992–1996, the coordination center of EPIC-Spain being located in Barcelona. All were mostly blood donors and volunteers. Details of the design and methodology of the EPIC and EPIC-Spain study have been described elsewhere [25,26]. Approval for the study was obtained from the ethical review boards of the International Agency for Research on Cancer and from the Medical Ethical Committee of Bellvitge Hospital (Barcelona, Spain). At the time the study sample was recruited, ethical protocol codes were not requested. Also, participants participated voluntarily in the study; all agreed to participate and to provide information on lifestyle and other variables for the study.

In order to account for a north-south dietary gradient, we considered participants from the two aforementioned EPIC-Spain centers. Both comprised 16,296 participants (N of EPIC-Granada = 7879 and N of EPIC-Gipuzkoa = 8417). Three hundred twenty-five individuals with extreme values of energy intake, below the first percentile (836 kcal/day) and above the 99th percentile (4119 kcal/day) of the distribution of energy intake, respectively, were excluded. Also, 1215 participants with prevalent diseases at baseline were excluded, leaving 14,756 healthy participants available for analysis.

For the biomarker study we considered subsample of 210 participants with complete information on diet, lifestyle and biomarker data. This sample was randomly chosen (by strata of sex, age and center) from the study sample. These participants were non users of dietary supplements and of drugs known to prompt OS, as well as participants who provided fasting blood samples. More details on the selection of these participants are provided elsewhere [21].

### 2.2. Data Collection: Dietary and Lifestyle Factors Assessment

The participants of both centers provided information about their dietary intake by means of a validated diet history questionnaire [27]. The questionnaire was administered through in-person interviews to collect information on the intake of more than 600 food items from the previous year. Seasonal variations in food consumption as well as other dietary issues, such as use of fats in the preparation of meals and consumption of alcoholic beverages were considered [25,27]. Intake of nutrients, along with total energy intake, were derived from the EPIC Nutrient Database-EPIC food composition data tables [28].

More details on how the dietary antioxidant profile was derived (vitamins, TAC, total polyphenols, flavonoids, lignans and other diet components) in the EPIC study can be found in Hernández-Ruiz, et al. 2018 [29]. In short, this information was derived from the EPIC-ENDB database, TAC values in food, and the Phenol Explorer database [18,28,30,31]. The Polyphenol Antioxidant Content (PAC) score was calculated from seven different polyphenols [30].

Participants were also asked to provide information about lifestyle habits, including smoking status (never, former, and current smoker), and smoking habits among smokers (intensity and duration of smoking). Anthropometric measurements (height, weight, waist and hip circumference) were also undertaken using standard protocols in all centers [31]. Body mass index (BMI) was calculated as weight in kilograms divided by the square of height in meters. According to WHO and ATP III cut-off points [32,33], we classified subjects into normal weight (BMI < 25 kg/m^2^), overweight (BMI ≥ 25 kg/m^2^) and obese (BMI ≥ 30 kg/m^2^) participants, and into normal waist circumference (WC) and abdominal obese (≥102 cm and ≥88 cm, in men and women, respectively) participants.

Regarding PA, information on occupational and leisure activities was collected through a validated PA questionnaire. This information was used to derive PA levels as inactive, moderately inactive, moderately active, and active (in metabolic equivalent units) [34].

### 2.3. Oxidative Balance Score (OBS) Components and Their Integration into Nutrient, Lifestyle and Food-Based OBSs

Two Oxidative Balance Scores (OBSs) and their simplified versions were developed. Overall, the OBSs were created so that higher scores could relate to predominance of antioxidant exposure. In particular, these scores were: 1) the Nutrient-Lifestyle OBS (NutrientL-OBS), 2) the Food-Lifestyle OBS (FoodL-OBS), and their simplified versions with dietary factors or lifestyle factors only: the Nutrient-based OBS (Nutrient-OBS), the Lifestyle OBS (L-OBS), and the Food-based OBS (Food-OBS). Supplementary Table S1 shows a summary of components considered in each OBS. All dietary, lifestyle and food components were selected due to their potential antioxidant and pro-oxidant components, considering their impact on the individual´s oxidative/antioxidant status. The rationale for their inclusion in these OBS has been provided in a review on applications and methods of OBSs [22]. Likewise, regarding the food-based OBS that we propose in this study, a summary as of why these components were chosen can be found in Supplementary Table S2.

*The NutrientL-OBS*: As aforementioned, its development has been based on the most widely applied concept of OBSs in the literature [22]. This NutrientL-OBS included 14 dietary and lifestyle components, namely: dietary antioxidant components (vitamin C, β-carotene, vitamin E (α-tocopherol), three global antioxidant potential measures (TRAP and FRAP, representing dietary TAC, and the PAC score); dietary pro-oxidants components (polyunsaturated fatty acids and heme-iron), some lifestyle antioxidants components (PA) and lifestyle pro-oxidants components: obesity in terms of BMI and WC, alcohol consumption, smoking habits and excess energy intake, this being the difference between the reported total dietary intake and the estimated total energy requirement.

All dietary variables were categorized into quintiles according to their distribution in the study population; for antioxidants (presumed to counteract OS), subjects who were in the lowest quintile of intake (quintile 1), were assigned 1 point, those who were in quintile 2, 2 points, and so forth. At the highest intake category (quintile 5), the maximum score of 5 points was awarded. Concerning the components with pro-oxidant potential (presumed to induce OS), the score was reversed, i.e., the lowest intake (quintile 1) was considered the least pro-oxidant and participants received 5 points, and so forth, until reaching the highest intake level (quintile 5) where participant got 1 point. Thus, these nutrient components were unweighted in the overall score because their contribution to the individual´s oxidative/antioxidant status was considered equally important. This view is based on previous nutrient-based OBSs [22]. The lifestyle factor components were PA, obesity, smoking and energy intake. Their scoring was formulated as follows:

- for PA, there were 3 categories established: sedentary/inactive = 1 point; moderately active = 3 points; active = 5 points;
- for alcohol, 1 to 5 points were assigned according to sex-specific levels of its consumption (men who consumed: ≤10 g/day = 5 points, ≤20 g/day = 4 points, ≤50 g/d = 3 points, ≤75 g/d = 2 points, and >75 g/d = 1 point, and women who consumed: ≤5 g/d = 5 points, ≤15 g/d = 4 points, ≤25 g/d = 3 points, ≤50 g/d = 2 points, and >50 g/d = 1 point;
- for overweight and obesity the assessment was made by classifying the subjects into 5 categories according to criteria established by the WHO and the Spanish Society for the Study of Obesity (SEEDO) [32,35]: <25 kg/m^2^ = 5 points), ≤ 26.9 kg/m^2^ = 4 points, ≤29.9 kg/m^2^ = 3 points, <35 kg/m^2^ = 2 points, and ≥ 35 kg/m^2^ = 1 point. Similarly, for abdominal obesity, following ATP III [33], the following points were assigned: men up to 102 cm of WC = 5 points, and 1 point otherwise, and women up to 88 cm = 5 points, while 1 point was given in the opposite case.
- for smoking, participants were classified as non-smokers = 5 points, former smokers = 3 points, or current smokers = 1 point. for excessive energy intake, when the reported intake was close to estimated energy expenditure = 5 points, if the intake did not exceed 10% of the requirement = 4 points, if it exceeded up to 20% = 3 points, for 30% of excessive intake= 2 points, and if it was above this amount = 1 point. The estimated energy expenditure was calculated beforehand as the amount of energy needed to maintain essential body functions plus the amount required to support the daily PA [36].

Finally, the NutrientL-OBS was calculated by adding the points assigned to each component, of both antioxidant and pro-oxidant factors. This score ranged from 14–70 points.

*The FoodL-OBS*: This OBS was developed to further to replace dietary aspects of the NutrientL-OBS by foods. The foods selected were those that contribute most notably to the dietary TAC. Pro-oxidant foods with a high content of pro-oxidant nutrients were also considered. Overall, there were 11 food components included in this OBS: 6 antioxidant-like food components (vegetables, fruits and juices, legumes, olive oil, blue or fatty fish and coffee and tea) and five pro-oxidant-like food components (meats and meat products, biscuits and pastries, fats and oils-except olive oil, snacks and sauces, cereals, and derived products, refined, roasted and fried). Other potential anti-oxidant foods (e.g., whole grains or nuts) were not considered due to the very low consumption pattern in this study population. On the other hand, the lifestyle factors components considered in this OBS were those included in the NutrientL-OBS.

Again, each component was divided into quintiles. The assignment of points was carried out as described above: in the case of foods rich in antioxidants, the lowest consumption quintile (quintile 1) received 1 point, the second quintile of lower consumption (quintile 2), received 2 points, and so forth, up to the quintile with the highest consumption (quintile 5), where 5 points were assigned. For pro-oxidant components, the scores were also reversed. In order to account for differences arising from the antioxidant potential of the food components, we considered to weight the components according to their antioxidant/pro-oxidant power. Specifically, we halved the points for those antioxidant components with intermediate antioxidant potential according to experimental TAC values (legumes, olive oil, fatty fish, cookies and pastries, snacks and sauces, cereals and products) [18,37]. Thus, this scoring system was the same as described above for the NutrientL-OBS, except that some food components were somehow weighted regarding their antioxidant potential. This FoodL-OBS considered 17 components in total, including 11 food/food groups components and six lifestyle components, the total score reaching a maximum score of 70 points.

*The simplified OBS versions*: Two modified versions of the NutrientL-OBS were developed: one that included dietary components only (8 components), the Nutrient OBS (Nutrient-OBS) and another one that included lifestyle components only (6 components), the Lifestyle OBS (L-OBS). The maximum scores reached in these modified OBSs were 40 and 30 points, respectively. Furthermore, we developed the lifestyle and food-based OBS (FoodL-OBS) in order to adjust components more easily. This OBS included 6 lifestyle components and 11 food components; the latter were separated into another OBS made up of food components only (Food-OBS).

### 2.4. Validation Study of the Oxidative Balance Scores: Relationship with Scores of Adherence to the Mediterranean Diet and with Biomarkers

Two validation studies were carried out to prove that the OBSs were effective tools to capture the individual´s oxidative and antioxidant status: (i) a validation study using scores of adherence to the Mediterranean Diet (MD), a dietary pattern rich in powerful antioxidants [29]; and, (ii) a validation study using biomarkers of OS, inflammation and antioxidant nutrients. The purpose was to verify whether higher values in the OBSs (towards more antioxidant-like states) were related to a higher adherence to the MD (towards a more antioxidant-like dietary pattern). In fact, a higher adherence to the MD, i.e., higher values in the scores, have been related to a higher intake of dietary nutrient antioxidants [29]. In this same line of thought, we sought to examine whether higher OBSs values were related to higher plasma levels of antioxidants. Given the link between OS and inflammation [6,38], we also considered inflammation and OS markers in this biomarker-based validation study. In this case, we hypothesized that a negative relationship exists between the OBSs and these markers (i.e., higher values of OBS related to lower levels of OS and inflammation markers).

For the validation study comprising the MD scores, the complete study sample of 14,756 subjects was used. In addition, we considered a total of 20 MD scores; all were previously applied in the EPIC Granada and Gipuzkoa study in a comparative study of their antioxidant profile [29,39].

For the validation study based on the biomarkers, we considered the subsample of 210 participants. As described previously [21], a set of biomarkers were quantified in fasting blood samples (plasma) that were collected at recruitment. These biomarkers were: (a) antioxidant vitamins (ascorbic and dehydroascorbic acid, as well as total vitamin C, α-tocopherol, β-carotene, vitamin A (retinol); coenzymes (coenzyme Q9 and Q10); (b) TAC: TRAP, FRAP and FRAP without uric acid, TEAC-ABTS, PT, ORAC with proteins and ORAC without proteins; (c) inflammation markers: C-reactive protein (CRP), Interleukin 6 and 8 (IL-6, IL-8) and Tumor necrosis factor alpha (TNF-α); (d) markers of oxidative stress (uric acid) and metabolic risk (adiponectin, plasminogen activator inhibitor-1 PAI-1, and resistin). The details of laboratory methods used and quality controls applied to each biomarker are provided in Carrión-García, et al., 2020 [21]. In brief, the determination of the TAC of the plasma samples was carried out with the methods of TRAP [40], FRAP [41], TEAC-ABTS [42], and ORAC [43]. The total polyphenols were determined by the Folin-Ciocalteu method [44]. Antioxidant capacity was expressed as μmol Trolox Equivalent/L of plasma. To rule out the possible influence of proteins on the TAC in terms of FRAP [19], the concentration of uric acid (~60% contribution to FRAP), measure of non-uric FRAP (FRAP–2*uric acid) was also considered. Likewise, the influence of proteins was accounted for in the ORAC assays. Biomarkers of antioxidant nutrients were determined by ultra-high-performance liquid chromatography (UHPLC) and mass spectrometer Acquity UHPLC BEH system. Biomarkers of inflammation were determined using Luminex technology. Enzyme-linked immunosorbent assay (ELISA) techniques were used to assess uric acid. In addition, CRP were quantified made using immunoturbidimetric determination. In all determinations, duplicate measurements were considered to estimate the intra- and inter-test coefficient of variation. This coefficient was less than 5% in the case of TAC, TP, and antioxidant nutrients, and less than 10% in the case of uric acid, CRP, and all other markers. While we measured other biomarkers of OS and inflammation (for example, oxidized LDL, malondialdehyde and IL-1), due to excessive variability in the measurements, we discarded these markers in this study.

### 2.5. Statistical Analyses

All OBSs were considered as a continuous variable and as a categorical variable, according to tertiles (T): lower score = T1, medium score = T2 and higher score = T3. Descriptive analyses were carried out by sex, centre and other characteristics of the study sample. The normality of all variables (kurtosis, skewness, and Shapiro–Wilk test) was studied, and a logarithmic transformation was performed on variables that did not show a normal distribution. Descriptive statistics were based on frequencies and percentages for categorical variables and means and standard deviation for continuous variables (with normal distribution). In addition, OBS scales and categories were compared by type of components (dietary, lifestyle and foods), scores of adherence to the MD and by plasma levels of antioxidants, inflammation and OS markers. To assess the differences between the groups (by sex and center, and by OBS tertiles; i.e., by two or three groups), we considered the t-Student or ANOVA tests (continuous variables, normal distribution), and Mann-Whitney U or Kruskal-Wallis tests (non-normal distribution), to compare two or more than two groups, as well as the Chi-squared test for categorical variables.

Non-parametric Spearman correlation analyses between all continuous variables were performed to study the direction and magnitude of the associations between the NutrientL-OBS, the dietary and lifestyle factor components, and the biomarkers. Heat map charts were created to visualize these correlations by the rho coefficients.

Different multivariate linear regression models were considered to examine the relationship between the different OBSs (X: independent variable) and a set of 20 MD scores (Y: dependent variable) [29]. Since it is necessary for a dependent variable to be normally distributed in linear regression models, we considered log-transformed biomarker and MD score variables. The models were defined as:

$$Log\left(biomarkers\;or\;MD\;scores\right)=\beta_{0}+\beta_{1}{\left(OBS\right)}_{1}+\beta_{2}X_{2}+\beta_{3}X_{3}\ldots +\varepsilon$$

The beta (β) coefficient, 95% confidence intervals and R2 determination coefficient were derived from these models. The OBSs were considered either as continuous variable or as categorical variable, the first tertile being the reference category. Thus, β coefficient were interpreted per increments of one unit in the OBS score (towards a more antioxidant status) or by the second or third tertile compared to the first tertile, respectively. A first model adjusted for age, sex, and centre, was considered. A second model was further adjusted for total energy intake. In a third model we adjusted for lifestyle factors involved in OS, namely BMI in kg/m^2^, smoking status and PA levels. Particularly, this third model was considered in those OBS that were based on dietary factors only (Nutrient-OBS and Food-OBS, without the lifestyle components) to better understand the effect of these lifestyle components on the associations, whereas the first and second models were considered in all OBS.

To assess the potential modifying effect of sex and center on these associations, models with and without a term of interaction (variable*sex or center) were considered, evaluating whether there were statistically significant differences between the two models through the likelihood ratio test. Stratified analyses by sex and center were also performed. Similarly, multivariate linear regression models were also applied to examine the association between the OBSs and the biomarkers (Y: dependent variable). All independent variables were log-transformed to approximate a normal distribution.

Given that we lacked values on some biomarkers, we conducted the analyses on the complete data, which is known to lead to bias estimates when the data are not missing completely at random [45]. The missing biomarker data in our study can be considered missing at random (MAR); for instance, the study center was related to the MAR pattern (*p* < 0.05). The missingness proportions varied from 0.3% (in plasma TAC) to 40% (in β-carotene). Imputation of missing data is very convenient even for relatively high proportions of missing data [46]. We decided to not impute missing values of α-carotene due to excess in missing data (90%). Thus, we applied multiple imputation by chained equations (MICE) to substitute the missing values [47]. We used predictive mean matching in a multivariate model including sex, age, center, bath, BMI and PA as covariates, with 10 iterations.

As a sensitivity analysis, to check the robustness of the results, the influence of extreme values and influential points of the biomarkers on the results obtained was evaluated. These points were identified by calculating Cook´s distances of each biomarker in the multivariate linear regression models, and comparing the results obtained after excluding these points [48]. Also, we compared results obtained in the biomarker analysis by complete-case analysis and by the imputed data. Finally, we verified that assumptions of linear regression models (linearity, homoscedasticity, normality, and absence of multicollinearity) were met (data not shown).

Statistically significant results were regarded as those *p*-values < 0.05. To account for multiple testing issues, we considered the Bonferroni corrected *p*-value threshold (0.05/20 MD sores or 25 biomarkers: 0.025 and 0.002, respectively). All analyses were performed using R software version 3.5.1 (R Core Team. R: a language and environment for statistical computing. R Found. Stat. Comput. 2018. http://www.r-project.org/, last accessed on 15 December 2021). Some of the R packages used were: mice, car, outliers, Hmisc and heatmaps.

## 3. Results

Below we describe the results regarding the characteristics of the different OBSs developed within this study, and of the validation study.

### 3.1. Characteristics of the Study Sample by the NutrientL-OBSs and FoodL-OBS

Table 1 shows characteristics of the study sample by sex and center, and according to the components of the NutrientL-OBS. There were 72% of men in EPIC-Gipuzkoa, and 27.7% in EPIC-Granada. The average age was 51 years in men and 49 years in women. Energy intake was significantly lower in women (1779 kcal/d) than in men (2521 kcal/d) (*p*-value < 0.001). Regarding the distribution in quintiles or categories of the components of this OBS, statistically significant differences were found between men and women in all components (*p*-value < 0.001). The most notable differences between men and women were observed for the alcohol and heme-iron component. By center, there were also statistically significant differences in the distribution of all the components (*p*-value < 0.001). For instance, the EPIC-Gipuzkoa center presented a higher proportion of subjects classified in the quintile scoring highest for the vitamin and TAC components. By contrast, the EPIC-Granada center received higher scores in the components of alcohol consumption, smoking habits, and that of excessive energy intake, than the EPIC-Gipuzkoa center. Also, participants of the EPIC-Gipuzkoa center were more physically active and had lower BMI and WC, than those of the EPIC-Granada center.

The overall NutrientL-OBS score was 48.6 points for men and 45.2 for women, this difference being statistically significant (*p*-value < 0.001). A higher proportion of men were classified in T3 (40.3%), while women were classified more frequently in T1 (41.9%). The average score was also significantly higher (*p*-value < 0.001) in the EPIC-Gipuzkoa center (47.7 points) with respect to the EPIC-Granada center (45 points). In fact, EPIC-Granada presented more participants in the first tertile compared to EPIC-Gipuzkoa (43.6 vs. 25.8%, *p*-value < 0.001).

Supplementary Table S3 shows the characteristics of the study population and components across tertiles of NutrientL-OBS (low, medium, and high scores). Subjects in the tertile of greater adherence (T3) consumed more energy (2278 kcal/d) and were younger (mean age: 48.6 years) than those classified in T1 (1798 kcal/d and 50.9 years) (*p*-value < 0.001). The mean score in every tertile was 38.7, 47 and 54.5, respectively.

Regarding the FoodL-OBS, as shown in Supplementary Table S4, there were also statistically significant differences between men and women in the food components (*p*-value < 0.001). Men were more frequently in the fifth quintile of intake of these foods than women, except for coffee and tea, meats, snacks and sauces, cereals and derived products. However, no statistically significant differences were observed by sex with respect to the average score (*p*-value = 0.944) or tertile classification (*p*-value = 0.286). By center, statistically significant differences were also found in almost all components (*p*-value < 0.001). Adherence to the FoodL-OBS was higher in EPIC-Gipuzkoa (50.7 points) than in EPIC-Granada (49.5 points), this difference being statistically significant (*p*-value < 0.001).

Descriptive statistics by tertiles of adherence to the FoodL-OBS (Table 2), showed that in the highest tertile (T3) there was a higher contribution of antioxidant foods (vegetables, fruit-juices, olive oil, coffee, and tea), while lower in the case of pro-oxidant foods (biscuits and pastries, oils and fats, snacks, and sauces). The mean scores varied from 43.5 points to 57.3 points across the tertiles. Dietary intakes of nutrients and foods of the NutrientL-OBS and FoodL-OBS by tertiles of adherence to these scores are shown in Supplementary Tables S5 and S6. Dietary antioxidant components increased significantly from the first to the third tertile, whereas an opposite trend was seen for the pro-oxidant components.

Overall, similar results were seen in the simplified versions of the OBSs (data not shown). The distribution of the components and OBSs were also very similar in the subsample of 210 subjects (data not shown).

### 3.2. Correlations between the OBSs, the MD Adherence Scores and the Biomarkers

**Figure 1:**
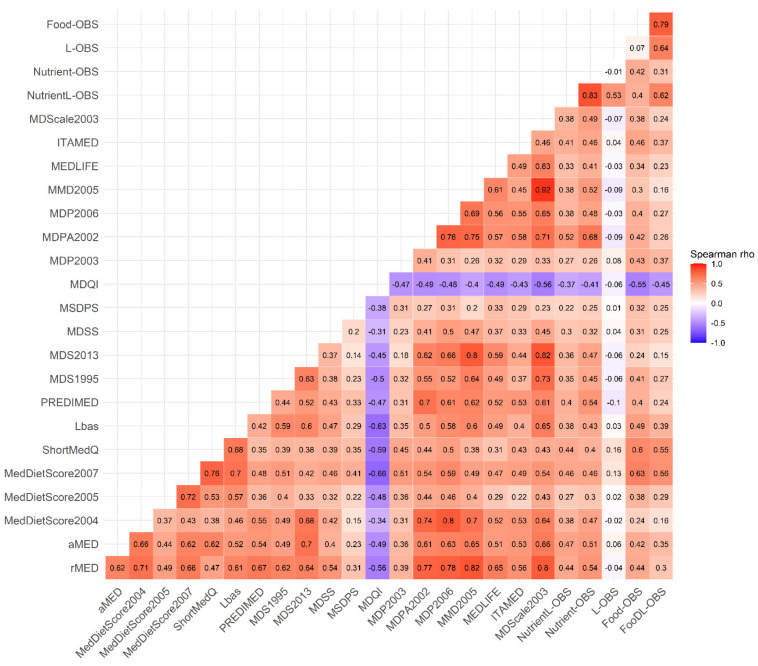
Correlation matrix between the Nutrient-Lifestyle and Food Oxidative Balance Score (NutrientL-OBS, Nutrient-OBS, L-OBS, FoodL-OBS and Food-OBS) and the 20 scores of adherence to the MD [29], in the EPIC Granada-Gipuzkoa study (N = 14,756). The color value of the cells is proportional to the strength of the associations, ranging from red (positive correlations) to blue (negative correlations), as indicated in the color scale (at the right of the panel). Pair-wise spearman correlation coefficients (rho) are shown in every cell. Correlations above 0.3 were all statistically significant (*p* < 0.05). Abbreviations: aMED: the Alternate MD Index; rMED: the Relative MD Score; MedDietScore: MD Score-2004, MD Score-2005, MD Score-207; ShortMedQ: the Cardioprotective MD Score; Lbas: the literature-based adherence score to the MD; PREDIMED: the Mediterranean food pattern of the PREDIMED Study (Mediterranean Diet Adherence Screener, MEDAS); MDS: the MDS-1995, MDS-2003; MDSS: Mediterranean Diet Serving Score; MSDPS: Mediterranean-Style Dietary Pattern Score; MDQI: the MD Quality Index; MDP: the Mediterranean Dietary Pattern-2002; Mediterranean Dietary Pattern-2006; MMD2005: the Modified MD-2005; MEDLIDE: the Mediterranean Lifestyle Index; ITAMED: Italian Mediterranean Index; MDScale2003: MDScale-2003; NutrientL-OBS: Nutrient-Lifestyle OBS; Nutrient-OBS: Nutrient-OBS; L-OBS: Lifestyle-OBS; FoodL-OBS: Lifestyle Food-OBS; Food- OBS: Food-OBS. MDQI: scoring goes in the opposite way (high refers to low adherence).

Figure 1 shows the strength of the association between the OBSs, the scores of adherence to the MD and the biomarkers, according to Spearman correlation analysis. The NutrientL-OBS and the FoodL-OBS and their simplified versions were positively correlated between each other (rho = 0.61 between the NutrientL-OBS and the LFood-OBS, and rho = 0.42 between the Nutrient-OBS and the Food-OBS), and were associated in a positive and statistically significant way with the majority of the MD scores (*p*-value < 0.05 for rho > 0.3), mainly with ITAMED MDPA02, PREDIMED, rMED, aMED, MDS95 and Lbas (rho = 0.4 to 0.7 for Food-OBS and Nutrient-OBS), and negatively with the MDQI score (rho = −0.55 and −0.41 for Food-OBS and Nutrient-OBS, respectively). Correlations became less strong when OBS with lifestyle components were considered. In fact, correlations with the MD scores were absent for the OBS made up of lifestyle components only (L-OBS).

**Figure 2:**
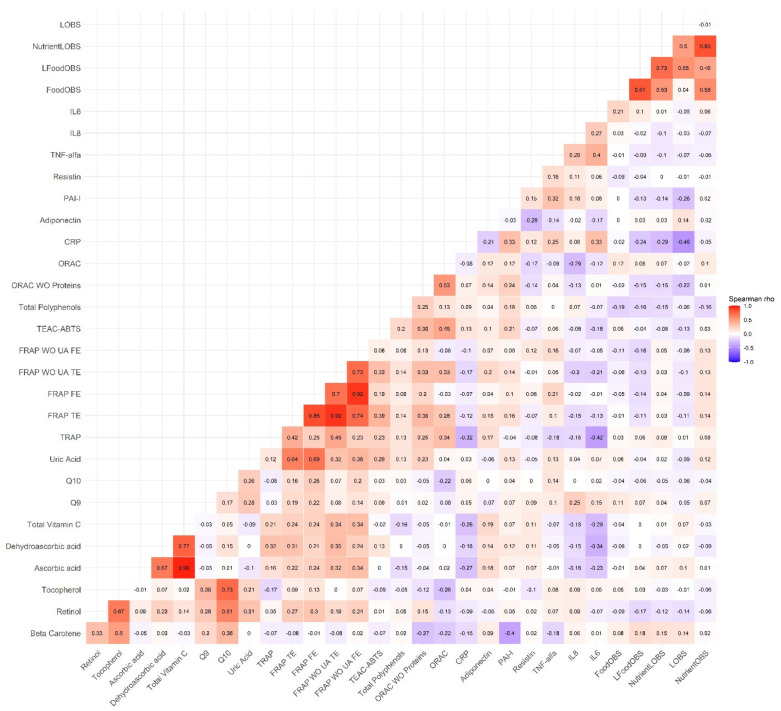
Correlation matrix between the Nutrient-Lifestyle and Food-Oxidative Balance Score (NutrientL-OBS, Nutrient-OBS, L-OBS, FoodL-OBS and Food-OBS) and the biomarkers of nutrient antioxidants (complete data), OS and inflammation, in the EPIC Granada-Gipuzkoa subsample (N = 210). The color value of the cells is proportional to the strength of the associations, ranging from red (positive correlations) to blue (negative correlations), as indicated in the color scale (at the right of the panel). Pair-wise spearman correlation coefficients (rho) are shown in every cell. Correlations above 0.3 were all statistically significant (*p* < 0.05). Abbreviations: WO = without; UA = uric acid; OS = oxidative stress; CRP: C-reactive protein; PAI-I: Plasminogen activator; TNF-α: tumor necrosis factor; IL = Interleukin; TRAP: total radical-trapping antioxidant parameter; FRAP: ferric-reducing antioxidant power; TEAC-ABTS: trolox equivalent antioxidant capacity—Azino Bis Thiazoline Sulfonic; ORAC: oxygen radical absorbance capacity; TE: Trolox equivalents; FE: iron equivalents.

Within the subsample of 210 participants (Figure 2), correlations between the different OBS were also strong/moderate (rho for NutrientL-OBS and FoodL-OBS = 0.71). Although the correlations were low, there were negative relations between the different OBS and some biomarkers of inflammation (TNF-α and CRP, rho = −0.3 to −0.4; PAI-I, rho = −0.2), while positive with adiponectin (rho = 0.3 to 0.4). With respect to nutrient antioxidants biomarkers, we observed positive modest correlations with Vitamin C and its derivatives (rho~0.3), TRAP (rho~0.3), and ORAC with proteins (rho~0.2). A negative correlation, through weak, emerged with total polyphenols (rho~−0.2). Null correlations were seen for other biomarkers. Interestingly, the above correlations were present for all the OBSs. It is also remarkable that the L-OBS, i.e., the lifestyle OBS without dietary factors, correlated more strongly with the inflammation makers (e.g., rho = 0.4 with adiponectin), whereas correlations with antioxidant nutrient makers seemed to be weaker and even tended to be negative with the TAC assays.

### 3.3. Linear Relationships between the OBSs, the MD Adherence Scores and the Biomarkers

Table 3 shows results from the multivariate linear regression analysis on the association between the NutrientL-OBS and the MD adherence scores adjusted for age, sex center, and energy intake. Concerning the NutrientL-OBS, all associations were positive and statistically significant (*p* < 0.001, after multiple testing correction); i.e., per one-unit increase in the OBS scoring, there was a significant increase in the adherence of the MD, the average increase being 0.02 points (on the log scale). The largest increases in MD adherence were observed for: aMED (β = 0.062), rMED (β = 0.023), MDQI (β= −0.023), MDS2013 (β = 0.029), MDS1995 and MDScale2003 (β = 0.03), shortMedQ (β = 0.024), MMD2005 (β = 0.028) and ITAMED (β = 0.028). Parellel to these associations, the percentage of the variance (i.e., the R2) that the independent variables (NutrientL-OBS and covariates) explained was higher than 15% for some MD scores (rMED, shortMedQ, PREDIMED, MDPA2002, MDQI and MedDietScore). This proportion of the variance ranged from 6% (MSDPS) to 51% (MDPA2002) in the most adjusted model. Adjustment for energy intake in Model 2 was almost negligible. When exploring FoodL-OBS and MD scores relations (Table 3), we observed similar results. As shown in Supplementary Tables S7 and S8, when the OBS were restricted to lifestyle (L-OBS) or the dietary components (Nutrient-OBS or Food-OBS), we found that the L-OBS was not consistently associated with the MD scores (*p*-values > 0.05 for rMED, MDS2013, MEDLIFE and others). In MD scores where associations reached statistical significance, the associations with the L-OBS were much weaker compared to those observed for the NutrientL-OBS or FoodL-OBS. By contrast, the associations with the MD scores turned stronger for the Nutrient-OBS and the Food-OBS, the latter showing more notable associations (for example, β = 0.027 with shortMedQ in Nutrient-OBS and β = 0.043 with shortMedQ in Food-OBS). Overall, the effect of the adjustment for energy intake on the associations was also minor. Adjustment for BMI, PA and smoking status did also not affect the β coefficient in these models.

Table 4 shows results regarding associations between NutrientL-OBS and FoodL-OBS with the biomarkers in the subsample. Similarly, Table 5 and Supplementary Table S9 show these associations concerning the simplified versions of these OBSs (Nutrient-OBS, Food-OBS and L-OBS). These results refer to the biomarker imputed data, i.e., missing values were replaced by the substituted ones. Higher NutrientL-OBS punctuations were associated with higher plasma levels (on the log scale) of β-carotene and of ascorbic acid (per one-unit increase in OBS: β = 0.02; *p*-value = 0.01, and β = 0.012; *p*-value = 0.02, respectively). In the same way, there was a positive association between the FoodL-OBS and ascorbic acid (β = 0.015; *p*-value = 0.008). Rather, there was a tendency towards an association between the FoodL-OBS with this and other nutrient antioxidant markers (for example, with retinol). Another striking finding was the inverse association between both NutrientL-OBS and FoodL-OBS with CRP (per one-unit increase in both OBS: β= −0.02; *p*-value = 0.02). While these associations did not meet the Bonferroni corrected *p*-value threshold (*p* = 0.002), these results sustain that a more pro-antioxidant state according to the DL-OBS is related to a higher antioxidant status. Surprisingly, there were also significant inverse associations between the NutrientL-OBS and FoodL-OBS with total polyphenols and uric acid, but these associations were not maintained after correction for multiple testing.

Concerning the associations between Nutrient-OBS and Food-OBS with the biomarkers (also on the log scale) (Table 5), we observed a trend towards positive associations between the Nutrient-OBS and ascorbic acid in plasma (*p*-value = 0.05), as well as with β-carotene (*p*-value = 0.1). Associations were statistically significant between the Food-OBS with ascorbic acid and total vitamin C (per one-unit increase in Food-OBS: β = 0.017; *p*-value = 0.018, and β = 0.017; *p*-value = 0.038, respectively). Adjustment for energy intake (model 1 vs. model 2) did also not have a notable influence on the estimates (data not shown). Interestingly, there was no association between these OBSs and CRP, suggesting that the removal of lifestyle components from these OBS led to this lack of association. Adjustment for lifestyle factors in model 3, indeed, did not affect the estimates (β coefficients) when compared to those derived from model 2. Moreover, the multivariate regression analyses between the L-OBS (i.e., the OBS made up of lifestyle factors only) and the biomarkers (Supplemental Table S7) revealed a strong inverse association between this OBS and CRP and PAI-I; per one-unit increase in L-OBS, these markers decreased on average (on the log scale) by 0.35 (*p*-value < 0.001) and 0.13 (*p*-value = 0.02), respectively. The proportion of variance explained by the predictors was also relatively high for this two markers (16–23%). However, opposite to what had been expected, negative associations were observed between this L-OBS and retinol, uric acid and FRAP (with and without uric acid). While associations between these simplified OBS versions (Nutrient-OBS, Food-OBS and L-OBS) and the nutrient antioxidant markers did not remain statistically significant after multiple testing correction, statistical significance was kept after this correction for the inflammation marker CRP.

### 3.4. Stratified and Sensitivity Analyses

We did not observe statistically significant interactions by sex (*p*-value = 0.30 for NutrientL-OBS, for example) or center (*p*-value = 0.11 for DL-OBS, for example). As a consequence, no changes in the results were observed in stratified analyses by these variables (data not shown). In analyses excluding influential values, prone to be outliers, results were also almost unchanged (data not shown). Results obtained in the multivariate regression analyses were similar when considering the unimputed biomarker data (data not shown). As shown in the correlation analyses, results derived from the imputed data (Supplementary Figure S1) was similar to those obtained from the complete data. Overall, similar results were obtained in these multivariate analyses when considering the OBS in tertiles as predictor variable (data not shown).

## 4. Discussion

In this study we propose two novel OBSs that are based on dietary and lifestyle factors (the NutrientL-OBS and FoodL-OBS) and some simplified versions thereof (the Nutrient-OBS, Food-OBS and L-OBS). The NutrientL-OBS implemented dietary and lifestyle components that were already considered in OBSs developed by others, and few new components related to OS that had thus far not been considered (for example, excess energy intake relative to the requirements, dietary TAC, and the PAC score). We also propose a novel food-based OBS, based on 11 anti- and pro-oxidant-like foods, to facilitate the assessment of the individual´s oxidative/antioxidant balance. Both OBSs were correlated with scores of adherence to the MD and with some biomarkers of nutrient antioxidants, inflammation and OS. More specifically, increasing scores in NutrientL-OBS and FoodL-OBS were both associated with increasing levels of certain nutrient antioxidants (ascorbic acid and Vitamin C), and with decreasing levels of inflammation markers such as CRP. An important finding to be highlighted is that the latter association seemed to be driven by the lifestyle components of these OBSs.

Earlier OBS reported in the literature included some relevant dietary and lifestyle factors related with OS [22]. Among them, few antioxidant and pro-oxidant factors were considered [24,49]. Further adaptations of these OBSs led to the inclusion of more factors [50,51]. However, improvements of these OBSs in order to better capture the oxidative/antioxidant balance can still be considered. In this study we proposed to consider dietary TAC, taking into account that TAC provides a holistic measure of the pool of antioxidants in the diet [19]. In fact, high dietary TAC has been associated with reduced mortality and lower risk of developing diseases [22,52,53,54,55] Specifically, two TAC methods (FRAP and TRAP) were included in the NutrientL-OBS and Nutrient-OBS so as to account for two different antioxidant mechanisms (proton or electron transfer). On the other hand, we also considered the PAC score, another measure of the global dietary intake of flavonoids and lignans [30]. Polyphenols have potential health benefits thanks to their association with OS, mostly by increasing the expression of antioxidant enzymes, by combating inflammation and by inhibiting cytotoxicity through intracellular regulation of calcium [56]. Also, we accounted for heme iron intake, which has been rarely considered in OBSs, despite its well-recognized pro-oxidant potential [57]. The rationale for the inclusion of other nutrient antioxidants (Vitamin C, retinol, etc.) in the OBSs has been provided in Hernández–Ruiz et al. 2019 [22]. Overall, our OBS incorporated in total eight dietary nutrient components.

In addition, six lifestyle components were considered, including those previously proposed by others [22]: PA, which is known to increase the adaptive response to OS by activating cellular antioxidant signaling systems [13,58], alcohol, which increases ROS generation by oxidizing ethanol to acetaldehyde [16,59], obesity, in terms of BMI, or WC, given its link with OS through lipid peroxidation processes [7], and tobacco consumption, which not only triggers oxidative imbalance between cell tissues, but also decreases the concentrations of some antioxidants in plasma while increasing inflammatory markers in blood and tissues [60]. We included BMI and waist circumference in the OBS to focus on both general and visceral obesity. In our OBS, we included additionally the estimated excess energy intake, i.e., the difference between energy intake and the individual’s energy requirements according to the basal metabolic rate and PA level. Indeed, an excessive energy intake contributes to the increase of OS since it triggers cellular stress [10]. Also, numerous experimental studies (in animals) have shown that caloric restriction reduces the risk of developing diseases associated with the aging process, or metabolic diseases such as type 2 diabetes mellitus [61,62,63].

Our study makes an important contribution to the development of OBS by creating one of the first food-based OBS. In the choice of foods as components of our OBS, their contribution to the dietary TAC has been considered from published TAC values of 210 foods [18,37], and other sources, as detailed in Supplementary Table S2. The degree of antioxidant or pro-oxidant potential of every food was considered. Therefore, some food components scored half as compared to other components. This consideration was taken into account to give the maximum score (positive or negative) to the components related to the intake of vegetables, fruits and juices, coffee and tea, meat and meat products. Due to low consumption patterns of some foods, we did not consider in this OBS consumption of nuts, whole grains or sweetened beverages, despite their high antioxidant or pro-oxidant capacity. To the best of our knowledge, in a recent study, a food-based OBS was proposed by accounting for the intake of 18 foods related to OS in a positive or negative manner [64]. Our food-based OBS contains 11 food components as several foods were grouped into certain components, to make its implementation easier. This lifestyle and food-based OBS also showed associations with nutrient antioxidant and inflammation makers, demonstrating likewise its capacity to assess the individual´s oxidative/antioxidant balance.

In the development of these OBSs we almost invariably applied equal weights to the components for the scoring. Studies that have adopted different weighting criteria to develop an OBS (for example, the OBS-Bayesian) showed similar results in studies addressing associations between these OBSs and disease risk [37,52,53]. The development of a biomarker-based OBS could better reflect the individual oxidative status. However, its use requires biomarker measurements in biological samples, which entails a high cost. OBSs based on dietary components estimated from diet questionnaires could also reflect antioxidant status, provided that they show a relationship with antioxidant and OS markers. Based on this, several OBSs have been validated in other studies concerning inflammation biomarkers [65,66,67] (for example, PCR) and OS [52,65,68] (for example, F2-isoprostanes, FIP). Recently, an OBS combining dietary and lifestyle factors weighted by associations with FIP, or unweighted, was also associated with this marker within the MAPI and MAPII study [64]. Together, these studies have demonstrated that well-defined OBS adequately reflect the individual oxidative/antioxidant balance. In this study, a validation study of our OBSs was also carried out. Our results also support that increasing OBSs scorings are related to higher levels of ascorbic acid and to lower inflammation levels of CRP, mainly. Ours is the first study showing associations between an OBSs with nutrient antioxidants and inflammation markers, that may be driven by dietary factors in the first case, and by lifestyle factors in the second. However, we did not observe associations with other biomarkers, such as plasma TAC. To date, it is unclear whether blood TAC levels are truly related to dietary intake of TAC. In fact, we have previously shown that there is a moderate positive correlation between diet and plasma TAC [69]. The unexpected negative relations between the OBS and uric acid, total polyphenols and other TAC measures were likely false and due to multiple testing problems. Another possible reason might be the influence of the enzymatic activity of the endogenous antioxidant defense system on these associations, for which we lacked information. In relation to uric acid we did not find a relationship with any of our OBSs, despite the recognized antioxidant power of this compound. Uric acid is a marker of OS according to findings of several studies showing that elevated uric acid levels in blood are associated with significantly decreased ROS levels [70,71]. On the other hand, uric acid at high levels or in certain media may have pro-oxidant properties [72]. This pro-oxidant nature could explain the inverse association between the OBS with uric acid in our study. Higher OBSs scores, however, showed positive trends towards higher levels of other antioxidant nutrients in plasma samples. These associations were not robust, possibly owing to the low bioavailability of these nutrients or the high inter-individual variability in measurements, amongst others. As has been reported in validation studies of dietary questionnaires, there is a moderate correlation between dietary intake of vitamin C and plasma levels of this vitamin, and a weak or no correlation for other vitamins [27]. The limited statistical power to observe these associations might have also affected these results.

Regarding the biomarker validation study using inflammatory markers, as aforementioned, we found inverse associations with CRP, supporting that high OBSs (towards more antioxidant states) can lower the levels of this marker. CRP is a widely studied biomarker of inflammation concerning CVD and other diseases related to inflammation and oxidative status [73]. No associations were found between the OBS with the other markers, except a positive tendency with adiponectin, a commonly used biomarker of obesity. In our study sample, for example, WC was negatively correlated with this marker, though moderately (rho = −0.34). We might have not observed an association due to this apparently low effect size or other issues. Adiponectin, produced by adipocytes, generates adipocytokines, which play a crucial role in metabolic and cardiovascular homeostasis. It is inversely related to OS and chronic inflammation, particularly with cytokines and chemokines such as TNF-alpha or IL-6 [74,75,76]. Therefore, it is likely that we did not observe associations with other makers. While there may be differences in the adiponectin/leptin relationship with obesity according to sex [77], no sex-modifying effects were observed in the associations evaluated.

The validation study of the OBSs developed in our study also comprised an examination of their relation with 20 scores of adherence to the MD [29]. Our OBSs were positively associated with all MD indexes, which supports that these OBSs also characterize an antioxidant-rich dietary pattern. The associations were stronger regarding some MD scores, suggesting that, while all DM indices evaluate the same diet pattern, there are not only differences in their antioxidant profile [29], but also in their association with the individual oxidative balance. It should be noted that these associations were most notable in OBSs that do not include lifestyle factors (Nutrient-OBS and Food-OBS), and absent for the L-OBS. This could be because MD score only include dietary components. However, in analyses controlling for lifestyle factors (PA, smoking habits, and BMI), we obtained similar positive associations between the OBSs and the MD scores.

Regarding limitations of this study, it is important to note that some nutrient components, such as selenium, zinc, omega-3 fatty acids, lycopene and lutein have not been included due to the lack of information on these components in the EPIC-ENDB database. Nor have the medication components such as aspirin and other non-steroidal anti-inflammatory drugs, or nutrient supplement intakes, been considered due to the lack of information in a large part of the study population. Also, the biomarker study might have been underpowered to observe robust associations between the OBS and markers. We must also note that this study did not cover relevant biomarkers of OS or those of the endogenous antioxidant defense system. Besides, the EPIC-Spain study counts with dietary data collected by means of a diet history questionnaire. While this method accounts for usual dietary intake that might not reflect immediate antioxidant intake, we have previously that shown dietary TAC assessed by this and other dietary assessment methods (24-h recall and FFQ) is similarly correlated to the non-enzymatic antioxidant capacity of the body that helps to relief the body from OS [21,69]. Among the strengths, it is important to highlight that we considered anthropometric parameters and information on PA i, that were measured in the EPIC study according to standard protocols. Ours is the first study showing associations between *a priori* defined OBSs combining nutrient, foods and lifestyle components with a wide range of MD scores and biomarkers of nutrient antioxidants, inflammation and OS. Moreover, our findings support that the different OBS link well to antioxidants in the diet and in the body, as well as to some inflammation-related markers, which validates their use for oxidative/antioxidant balance assessment. We were able to control for the influence of lifestyle factors on the associations, ruling out, to the extent possible, potential residual confounding on the studied associations. We had also extensive and high quality information on dietary intake of foods and nutrients, that allowed us to derive dietary factors such as the PAC scores and dietary TAC, for their inclusion in the different OBSs. Finally, we have explored in depth how nutrient and food-based OBS with lifestyle factors, and their simplified versions as only nutrient, food or lifestyle components, are related to variables of the individual´s oxidative and antioxidant status.

## 5. Conclusions

The current study proposes two new OBSs (the NutrientL-OBS and FoodL-OBS) that incorporate diet, lifestyle and food dimensions in a single score. Both OBSs are easy-to-implement due to their simplicity, and both can be considered valid tools to assess the individual´s oxidative/antioxidant balance, given their association with antioxidant-rich dietary patterns and with some biomarkers of nutrient antioxidants and inflammation. Our study also supports that OBSs need to rely on both dietary and lifestyle components in order to reflect the oxidative/antioxidant state of an individual. The proposed OBSs might be useful for future studies seeking to assess associations between OS induced by dietary and lifestyle factors and risk of developing diseases.

## Figures and Tables

**Table 1:** Description of the components included in the Dietary-Lifestyle Nutrient Oxidative Balance Score (NutrientL-OBS) in the EPIC Granada-Gipuzkoa study (N = 14,756) by sex (5517 men and 9239 women) and centre (6625 Granada and 8131 Gipuzkoa).

	Men		Women			Granada		Gipuzkoa		
	N	%	N	%	*p*-Value ^a^	N	%	N	%	*p*-Value ^a^
Eight Nutrient components of NutrientL-OBS (in quintiles, Q)										
Vitamin C, mg/d ^γ^					0.007					<0.001
Q1 (1 point)	1095	19.8%	1857	20.1%		1093	16.5%	1859	22.9%	
Q2 (2 points)	1044	18.9%	1907	20.6%		1462	22.1%	1489	18.3%	
Q3 (3 points)	1077	19.5%	1874	20.3%		1441	21.8%	1510	18.6%	
Q4 (4 points)	1172	21.2%	1779	19.3%		1345	20.3%	1606	19.8%	
Q5 (5 points)	1129	20.5%	1822	19.7%		1284	19.4%	1667	20.5%	
β-carotene, µg/d ^γ^					<0.001					<0.001
Q1 (1 point)	998	18.1%	1954	21.1%		1460	22.0%	1492	18.3%	
Q2 (2 points)	1079	19.6%	1872	20.3%		1456	22.0%	1495	18.4%	
Q3 (3 points)	1114	20.2%	1837	19.9%		1363	20.6%	1588	19.5%	
Q4 (4 points)	1139	20.6%	1812	19.6%		1214	18.3%	1737	21.4%	
Q5 (5 points)	1187	21.5%	1764	19.1%		1132	17.1%	1819	22.4%	
α-Tocopherol, mg/d ^γ^					<0.001					<0.001
Q1 (1 point)	523	9.48%	2429	26.3%		2028	30.6%	924	11.4%	
Q2 (2 points)	795	14.4%	2156	23.3%		1688	25.5%	1263	15.5%	
Q3 (3 points)	1066	19.3%	1885	20.4%		1348	20.3%	1603	19.7%	
Q4 (4 points)	1375	24.9%	1576	17.1%		1014	15.3%	1937	23.8%	
Q5 (5 points)	1758	31.9%	1193	12.9%		547	8.26%	2404	29.6%	
TRAP, µmol trolox ^γ^					<0.001					<0.001
Q1 (1 point)	460	8.34%	2492	27.0%		1836	27.7%	1116	13.7%	
Q2 (2 points)	631	11.4%	2320	25.1%		1669	25.2%	1282	15.8%	
Q3 (3 points)	878	15.9%	2073	22.4%		1399	21.1%	1552	19.1%	
Q4 (4 points)	1331	24.1%	1620	17.5%		1128	17.0%	1823	22.4%	
Q5 (5 points)	2217	40.2%	734	7.94%		593	8.95%	2358	29.0%	
FRAP, µmol iron/d ^γ^					<0.001					<0.001
Q1 (1 point)	407	7.38%	2545	27.5%		1772	26.7%	1180	14.5%	
Q2 (2 points)	620	11.2%	2331	25.2%		1615	24.4%	1336	16.4%	
Q3 (3 points)	924	16.7%	2027	21.9%		1439	21.7%	1512	18.6%	
Q4 (4 points)	1382	25.0%	1569	17.0%		1140	17.2%	1811	22.3%	
Q5 (5 points)	2184	39.6%	767	8.30%		659	9.95%	2292	28.2%	
PAC score, -28-28 ^γ^					<0.001					<0.001
Q1 (1 point)	485	8.79%	2609	28.2%		1760	26.6%	1334	16.4%	
Q2 (2 points)	719	13.0%	2396	25.9%		1660	25.1%	1455	17.9%	
Q3 (3 points)	993	18.0%	2007	21.7%		1402	21.2%	1598	19.7%	
Q4 (4 points)	1339	24.3%	1504	16.3%		1078	16.3%	1765	21.7%	
Q5 (5 points)	1981	35.9%	723	7.83%		725	10.9%	1979	24.3%	
PUFA, g/d ^≠^					<0.001					<0.001
Q1 (5 points)	1836	33.3%	1115	12.1%		467	7.05%	2484	30.5%	
Q2 (4 points)	1406	25.5%	1545	16.7%		1029	15.5%	1922	23.6%	
Q3 (3 points)	1176	21.3%	1775	19.2%		1396	21.1%	1555	19.1%	
Q4 (2 points)	747	13.5%	2204	23.9%		1732	26.1%	1219	15.0%	
Q5 (1 point)	352	6.38%	2600	28.1%		2001	30.2%	951	11.7%	
Heme-iron, mg/d ^≠^					<0.001					<0.001
Q1 (5 points)	2114	38.3%	837	9.06%		521	7.86%	2430	29.9%	
Q2 (4 points)	1425	25.8%	1526	16.5%		804	12.1%	2147	26.4%	
Q3 (3 points)	981	17.8%	1970	21.3%		1274	19.2%	1677	20.6%	
Q4 (2 points)	643	11.7%	2308	25.0%		1752	26.4%	1199	14.7%	
Q5 (1 point)	354	6.42%	2598	28.1%		2274	34.3%	678	8.34%	
Six Lifestyle factors components of NutrientL-OBS (in categories)										
PA, METs/d ^γ^					<0.001					<0.001
Inactive (1 points)	1132	20.5%	4604	49.8%		3486	52.6%	2250	27.7%	
Moderate (3 points)	3174	57.5%	4715	46.0%		2705	40.8%	4715	58.0%	
Active (5 points)	1211	22.0%	389	4.21%		434	6.55%	1166	14.3%	
Alcohol, g/d ^≠^					<0.001					<0.001
>75, M->50, W (1 point)	338	6.13%	29	0.31%		27	0.41%	340	4.18%	
≤75, M-≤50, W (2 points)	641	11.6%	325	3.52%		101	1.52%	865	10.6%	
≤50, M-≤25, W (3 points)	1855	33.6%	486	5.26%		516	7.79%	1825	22.4%	
≤20, M-≤15, W (4 points)	838	15.2%	1208	13.1%		681	10.3%	1365	16.8%	
≤10, M-≤5, W (5 points)	1845	33.4%	7191	77.8%		5300	80.0%	3736	45.9%	
BMI, kg/m^2 ≠^					<0.001					<0.001
≥35 (1 point)	180	3.26%	852	9.22%		688	10.4%	344	4.23%	
<35 (2 points)	1306	23.7%	2112	22.9%		1923	29.0%	1495	18.4%	
≤29.9 (3 points)	2006	36.4%	2135	23.1%		1793	27.1%	2348	28.9%	
≤26.9 (4 points)	1194	21.6%	1576	17.1%		1004	15.2%	1766	21.7%	
<25 (5 points)	831	15.1%	2564	27.8%		1217	18.4%	2178	26.8%	
WC, cm ^≠^					<0.001					<0.001
>102, M; >88, W (1 point)	1814	32.9%	3927	42.5%		3229	48.7%	2512	30.9%	
<102. M; <88, W (5 points)	3703	67.1%	5312	57.5%		3396	51.3%	5619	69.1%	
Smoking status ^≠^					<0.001					<0.001
Never (5 points)	1674	30.3%	6835	74.0%		4423	66.8%	4086	50.3%	
Former (3 points)	1679	30.4%	942	10.2%		996	15.0%	1625	20.0%	
Current (1 points)	2164	39.2%	1462	15.8%		1206	18.2%	2420	29.8%	
Excess energy intake, kcal ^≠^					<0.001					<0.001
Excess energy >30% (1 points)	296	5.37%	322	3.49%		170	2.57%	448	5.51%	
Excess energy <30% (2 points)	317	5.75%	292	3.16%		155	2.34%	454	5.58%	
Excess energy <20% (3 points)	485	8.79%	468	5.07%		270	4.08%	683	8.40%	
Excess energy <10% (4 points)	735	13.3%	769	8.32%		439	6.63%	1065	13.1%	
Similar intake (5 points)	3684	66.8%	7388	80.0%		5591	84.4%	5481	67.4%	
Distribution of the NutrientL-OBS										
NutrientL-OBS (by tertiles)					<0.001					<0.001
T1	1117	20.2%	3874	41.9%		2891	43.6%	2100	25.8%	
T2	2178	39.5%	3077	33.3%		2154	32.5%	3101	38.1%	
T3	2222	40.3%	2288	24.8%		1580	23.8%	2930	36.0%	

The mean score NutrientL-OBS was (mean points/SD): men (48.6/6.16), and woman (45.2/7,14), *p*-value < 0.001; in the EPIC-Granada center (45.0/7.17) and in the EPIC-Gipuzkoa center (47.7/6.56), *p*-value < 0.001. ^γ^ Antioxidant components; ^≠^ Pro-oxidant components. ^a^ The differences between groups have been assessed using the Chi-square test (categorical variables) or t-Student or U Mann Whitney test (continuous variables), depending on the distribution of the variable. Descriptives are shown as frequencies and percentages (continuous variables). Abbreviations: TRAP: Total Radical Trapping Antioxidant Parameter, FRAP: Ferric Reducing Antioxidant Power PAC Score: Polyphenol Antioxidant Content Score; BMI: Body Mass Index, WC: Waist Circumference, PA: Physical Activity, PUFA: Polyunsaturated fatty acids; M: Men, W: Women, Q: Quintiles, T = Tertiles.

**Table 2:** Characteristics of the components included in the Dietary-Lifestyle Food Oxidative Balance Score (FoodL-OBS) in the EPIC Granada-Gipuzkoa study (N = 14,756) by adherence tertiles.

	Tertile 1 (T1); N = 4917		Tertile 2 (T2); N = 4900		Tertile 3 (T3); N = 4939		
	N	%	N	%	N	%	*p*-Value ^a^
Eleven Food components of FoodL-OBS (in quintiles, Q)							
Vegetable, g/d ^γ^							<0.001
Q1 (1 point)	1644	33.4%	935	19.1%	373	7.55%	
Q2 (2 points)	1176	23.9%	1100	22.4%	675	13.7%	
Q3 (3 points)	928	19.0%	1042	21.3%	971	19.7%	
Q4 (4 points)	712	14.5%	988	20.2%	1251	25.3%	
Q5 (5 points)	447	9.09%	835	17.0%	1669	33.8%	
Fruits and juices, g/d ^γ^							<0.001
Q1 (1 point)	1636	33.3%	898	18.3%	418	8.46%	
Q2 (2 points)	1203	24.5%	1075	21.9%	727	14.7%	
Q3 (3 points)	901	18.3%	1018	20.8%	978	19.8%	
Q4 (4 points)	699	14.2%	1020	20.8%	1232	24.9%	
Q5 (5 points)	478	9.72%	889	18.1%	1584	32.1%	
Legumes, g/d ^γ.^							<0.001
Q1 (0.5 points)	1085	22.1%	955	19.5%	913	18.5%	
Q2 (1 point)	1021	20.8%	1010	20.6%	937	19.0%	
Q3 (1.5 points)	944	19.2%	1032	21.1%	1024	20.7%	
Q4 (2 points)	868	17.7%	965	19.7%	1058	21.4%	
Q5 (2.5 points)	999	20.3%	938	19.1%	1007	20.4%	
Olive oil, g/d ^γ^^.^							<0.001
Q1 (0.5 points)	1830	37.2%	786	16.0%	336	6.80%	
Q2 (1 point)	1175	23.9%	1095	22.3%	681	13.8%	
Q3 (1.5 points)	796	16.2%	1095	22.3%	1060	21.5%	
Q4 (2 points)	597	12.1%	995	20.3%	1359	27.5%	
Q5 (2.5 points)	519	10.6%	929	19.0%	1503	30.4%	
Fatty fish, g/d ^γ^							<0.001
Q1 (0.5 points)	1212	24.6%	945	19.3%	850	17.2%	
Q2 (1 point)	993	20.2%	1044	21.3%	831	16.8%	
Q3 (1.5 points)	1056	21.5%	924	18.9%	998	20.2%	
Q4 (2 points)	845	17.2%	1118	22.8%	988	20.0%	
Q5 (2.5 points)	811	16.5%	869	17.7%	1272	25.8%	
Coffee and tea, g/d ^γ^							<0.001
Q1 (1 point)	1252	25.5%	1018	20.8%	682	13.8%	
Q2 (2 points)	1168	23.8%	977	19.9%	810	16.4%	
Q3 (3 points)	1123	22.8%	1138	23.2%	1003	20.3%	
Q4 (4 points)	733	14.9%	850	17.3%	1055	21.4%	
Q5 (5 points)	641	13.0%	917	18.7%	1389	28.1%	
Meat and meat products, g/d ^≠^							<0.001
Q5 (1 point)	1490	28.0%	869	18.4%	592	12.6%	
Q4 (2 points)	1331	25.0%	920	19.5%	700	14.8%	
Q3 (3 points)	1052	19.8%	1001	21.2%	898	19.1%	
Q2 (4 points)	793	14.9%	935	19.8%	1223	25.9%	
Q1 (5 points)	656	12.3%	996	21.1%	1300	27.6%	
Cookies and pastries, g/d ^≠^							<0.001
Q5 (0.5 points)	1073	21.8%	836	17.1%	628	12.7%	
Q4 (1 point)	939	19.1%	856	17.5%	723	14.6%	
Q3 (1.5 points)	876	17.8%	878	17.9%	805	16.3%	
Q2 (2 points)	719	14.6%	859	17.5%	960	19.4%	
Q1 (2.5 points)	1310	26.6%	1471	30.0%	1823	36.9%	
Fats and oils, g/d ^≠^							<0.001
Q5 (1 point)	1596	30.0%	631	13.4%	218	4.63%	
Q4 (2 points)	1354	25.4%	741	15.7%	349	7.41%	
Q3 (3 points)	852	16.0%	875	18.5%	717	15.2%	
Q2 (4 points)	570	10.7%	832	17.6%	1043	22.1%	
Q1 (5 points)	950	17.8%	1642	34.8%	2386	50.6%	
Snacks and sauces, g/d ^≠^							<0.001
Q5 (0.5 points)	1554	31.6%	914	18.7%	483	9.78%	
Q4 (1 point)	1122	22.8%	990	20.2%	839	17.0%	
Q3 (1.5 points)	883	18.0%	1016	20.7%	1024	20.7%	
Q2 (2 points)	740	15.0%	961	19.6%	1276	25.8%	
Q1 (2.5 points)	618	12.6%	1019	20.8%	1317	26.7%	
Cereals and refined products, g/d ^≠^							<0.001
Q5 (0.5 points)	1543	31.4%	917	18.7%	491	9.94%	
Q4 (1 point)	1161	23.6%	1019	20.8%	770	15.6%	
Q3 (1.5 points)	944	19.2%	981	20.0%	942	19.1%	
Q2 (2 points)	764	15.5%	1047	21.4%	1224	24.8%	
Q1 (2.5 points)	505	10.3%	936	19.1%	1512	30.6%	
Six Lifestyle factors components of FoodL-OBS (in categories)							
PA, METs/d ^γ^							<0.001
Inactive (1 point)	2239	45.5%	2023	41.3%	1474	29.8%	
Moderate (3 points)	2289	46.6%	2388	48.7%	2743	55.5%	
Active (5 points)	389	7.91%	489	9.98%	722	14.6%	
Alcohol consumption, g/d ^≠^							<0.001
>75, M->50, W (1 point)	300	6.10%	55	1.12%	12	0.24%	
≤75, M-≤50, W (2 points)	600	12.2%	265	5.41%	101	2.04%	
≤50, M-≤25, W (3 points)	1101	22.4%	763	15.6%	477	9.66%	
≤20, M-≤15, W (4 points)	639	13.0%	713	14.6%	694	14.1%	
≤10, M-≤5, W (5 points)	2277	46.3%	3104	63.3%	3655	74.0%	
BMI, kg/m^2 ≠^							<0.001
≥35 (1 point)	596	12.1%	321	6.55%	115	2.33%	
<35 (2 points)	1663	33.8%	1172	23.9%	583	11.8%	
≤29.9 (3 points)	1401	28.5%	1408	28.7%	1332	27.0%	
≤26.9 (4 points)	616	12.5%	875	17.9%	1279	25.9%	
<25 (5 points)	641	13.0%	1124	22.9%	1630	33.0%	
WC, cm ^≠^							<0.001
>102, M; >88, W (1 point)	2988	60.8%	1937	39.5%	816	16.5%	
<102, M; <88, W (5 points)	1929	39.2%	2963	60.5%	4123	83.5%	
Smoking status ^≠^							<0.001
Current (1 point)	1860	37.8%	1092	22.3%	674	13.6%	
Former (3 points)	847	17.2%	893	18.2%	881	17.8%	
Never (5 points)	2210	44.9%	2915	59.5%	3384	68.5%	
Excess energy intake according to total energy expenditure component, kcal (categories)							<0.001
Excess energy intake >30% (1 point)	372	7.57%	178	3.63%	68	1.38%	
Excess energy intake <30% (2 points)	304	6.18%	189	3.86%	116	2.35%	
Excess energy intake <20% (3 points)	434	8.83%	293	5.98%	226	4.58%	
Excess energy intake <10% (4 points)	575	11.7%	486	9.92%	443	8.97%	
Similar energy intake (5 points)	3232	65.7%	3754	76.6%	4086	82.7%	

The score FoodL-OBS was (mean points/SD): T1 (43.5/3.11), T2 (50.5/1.7), and (57.3/2.94), *p*-value < 0.001. ^γ^ Antioxidant components; ^≠^ Pro-oxidant components. ^a^ The differences between groups have been assessed using the Chi-square (categorical variables). Descriptives are shown in frequencies and percentages for categorical variables. Abbreviations: BMI: Body Mass Index, WC: Waist Circumference, PA: Physical Activity, M: Men, W: Women, Q: Quintiles, T = Tertiles.

**Table 3:** Multivariate linear regression analysis between adherence to the Nutrient-Lifestyle and Food-Lifestyle Oxidative Balance Scores (NutrientL-OBS and FoodL-OBS) and the Mediterranean Diet scores (MD) in the Granada-Gipuzkoa EPIC study (N = 14,756).

	NutrientL-OBS										FoodL-OBS									
	Model 1					Model 2					Model 1					Model 2				
	β	95% CI		*p*-Value	R^2^	β	95% CI		*p*-Value	R^2^	β	95% CI		*p*-Value	R^2^	β	95% CI		*p*-Value	R^2^
**rMED**	0.023	0.022	0.023	<0.001	0.243	0.021	0.021	0.022	<0.001	0.288	0.023	0.022	0.024	<0.001	0.221	0.028	0.028	0.029	<0.001	0.342
**aMED**	0.062	0.058	0.065	<0.001	0.076	0.06	0.057	0.064	<0.001	0.079	0.064	0.06	0.068	<0.001	0.067	0.072	0.068	0.076	<0.001	0.087
**MedDietScore2004**	0.008	0.007	0.008	<0.001	0.259	0.007	0.007	0.007	<0.001	0.384	0.006	0.006	0.006	<0.001	0.194	0.009	0.008	0.009	<0.001	0.405
**MedDietScore2005**	0.006	0.005	0.006	<0.001	0.105	0.006	0.005	0.006	<0.001	0.113	0.007	0.007	0.008	<0.001	0.129	0.008	0.008	0.009	<0.001	0.169
**MedDietScore2007**	0.009	0.009	0.009	<0.001	0.251	0.009	0.009	0.01	<0.001	0.252	0.012	0.012	0.012	<0.001	0.344	0.013	0.013	0.013	<0.001	0.371
**ShortMedQ**	0.024	0.023	0.024	<0.001	0.172	0.024	0.023	0.024	<0.001	0.172	0.031	0.03	0.032	<0.001	0.226	0.033	0.032	0.034	<0.001	0.248
**Lbas**	0.014	0.013	0.014	<0.001	0.143	0.013	0.013	0.014	<0.001	0.151	0.016	0.015	0.016	<0.001	0.148	0.018	0.017	0.018	<0.001	0.197
**PREDIMED**	0.009	0.009	0.01	<0.001	0.241	0.009	0.008	0.009	<0.001	0.287	0.009	0.008	0.009	<0.001	0.205	0.011	0.011	0.011	<0.001	0.319
**MDS1995**	0.03	0.028	0.032	<0.001	0.058	0.029	0.027	0.031	<0.001	0.068	0.026	0.024	0.028	<0.001	0.035	0.032	0.03	0.034	<0.001	0.065
**MDS2013**	0.029	0.027	0.031	<0.001	0.084	0.027	0.026	0.029	<0.001	0.097	0.021	0.019	0.023	<0.001	0.054	0.027	0.025	0.029	<0.001	0.086
**MDSS**	0.012	0.011	0.012	<0.001	0.085	0.011	0.01	0.012	<0.001	0.107	0.012	0.012	0.013	<0.001	0.082	0.015	0.015	0.016	<0.001	0.143
**MSDPS**	0.006	0.006	0.007	<0.001	0.057	0.006	0.006	0.007	<0.001	0.057	0.008	0.008	0.009	<0.001	0.073	0.009	0.008	0.009	<0.001	0.08
**MDQI**	−0.023	−0.023	−0.02	<0.001	0.176	−0.02	−0.02	−0.02	<0.001	0.185	−0.028	−0.029	−0.027	<0.001	0.214	−0.03	−0.03	−0.03	<0.001	0.217
**MDP2003**	0.008	0.007	0.008	<0.001	0.096	0.008	0.008	0.008	<0.001	0.113	0.011	0.011	0.012	<0.001	0.165	0.011	0.011	0.012	<0.001	0.165
**MDPA2002**	0.014	0.014	0.015	<0.001	0.412	0.013	0.013	0.014	<0.001	0.515	0.012	0.011	0.012	<0.001	0.311	0.015	0.015	0.016	<0.001	0.529
**MDP2006**	0.008	0.008	0.008	<0.001	0.22	0.007	0.007	0.007	<0.001	0.362	0.008	0.008	0.008	<0.001	0.202	0.011	0.011	0.011	<0.001	0.458
**MMD2005**	0.028	0.026	0.03	<0.001	0.131	0.026	0.024	0.027	<0.001	0.171	0.024	0.022	0.026	<0.001	0.104	0.032	0.03	0.034	<0.001	0.181
**MEDLIFE**	0.009	0.009	0.01	<0.001	0.141	0.009	0.009	0.009	<0.001	0.147	0.009	0.009	0.01	<0.001	0.128	0.011	0.01	0.011	<0.001	0.161
**ITAMED**	0.028	0.026	0.029	<0.001	0.074	0.027	0.026	0.029	<0.001	0.075	0.033	0.031	0.035	<0.001	0.085	0.037	0.035	0.039	<0.001	0.099
**MDScale2003**	0.03	0.028	0.031	<0.001	0.101	0.029	0.027	0.03	<0.001	0.106	0.029	0.027	0.031	<0.001	0.089	0.034	0.032	0.036	<0.001	0.112

Mediterranean Diet scores (DM): aMED: the Alternate MD Index; rMED: the Relative MD Score; MedDietScore: MD Score-2004, MD Score-2005, MD Score-207; ShortMedQ: the Cardioprotective MD Score; Lbas: the literature-based adherence score to the MD; PREDIMED: the Mediterranean food pattern of the PREDIMED Study (Mediterranean Diet Adherence Screener, MEDAS); MDS: the MDS-1995, MDS-2003; MDSS: Mediterranean Diet Serving Score; MSDPS: Mediterranean-Style Dietary Pattern Score; MDQI: the MD Quality Index; MDP: the Mediterranean Dietary Pattern-2002; Mediterranean Dietary Pattern-2006; MMD2005: the Modified MD-2005; MEDLIDE: the Mediterranean Lifestyle Index; ITAMED: Italian Mediterranean Index; MDScale2003: MDScale-2003; MDQI: scoring goes in the opposite way (high refers to low adherence). Model 1: adjusted for age (continuous), sex and center. Model 2: additionally, adjusted for energy intake in kcal (continuous). All MD score (dependent variables) were log-transformed to approximate a normal distribution. The coefficients β, the corresponding 95% confidence intervals (CI) and R2 are shown (proportion of the variance explained by the independent variables). *p*-values threshold after multiple testing correction = 0.025. The largest β increments as well as the R2 that explain the highest and lowest variance are latticed. The coloured rows in grey show the models with the strongest associations (positive and negative).

**Table 4:** Multivariate linear regression analysis between adherence to the Nutrient-Lifestyle and Food-Lifestyle Oxidative Balance Score (NutrientL-OBS and FoodL-OBS) and the biomarkers of nutrient antioxidants (imputed data), OS and inflammation, in the EPIC Granada-Gipuzkoa subsample (N = 210). Imputed data.

	NutrientL-OBS										FoodL-OBS									
	Model 1					Model 2					Model 1					Model 2				
	β	95% CI		*p*-Value	R^2^	β	95% CI		*p*-Value	R^2^	β	95% CI		*p*-Value	R^2^	β	95% CI		*p*-Value	R^2^
**β-carotene**	0.02	0.004	0.035	0.012	0.078	0.02	0.005	0.036	0.010	0.086	0.012	−0.004	0.029	0.143	0.06	0.014	−0.002	0.031	0.096	0.069
**Retinol**	−0.004	−0.01	0.001	0.116	0.086	−0.004	−0.01	0.001	0.112	0.087	−0.005	−0.011	0.001	0.078	0.089	−0.005	−0.011	0.000	0.068	0.090
**Tocopherol**	−0.001	−0.008	0.007	0.876	0.01	−0.001	−0.009	0.007	0.827	0.015	0.002	−0.006	0.011	0.587	0.011	0.002	−0.007	0.01	0.691	0.015
**Ascorbic acid**	0.012	0.002	0.023	0.025	0.273	0.012	0.002	0.023	0.022	0.275	0.014	0.003	0.026	0.013	0.277	0.015	0.004	0.027	0.008	0.282
**Dehydroascorbic acid**	−0.017	−0.104	0.07	0.705	0.339	−0.015	−0.103	0.072	0.728	0.339	0.013	−0.08	0.105	0.787	0.338	0.017	−0.077	0.111	0.727	0.339
**Total Vitamin C**	0.011	−0.001	0.023	0.077	0.308	0.011	−0.001	0.023	0.065	0.313	0.013	0	0.026	0.049	0.311	0.014	0.001	0.027	0.030	0.318
**Q9**	0.004	−0.011	0.019	0.593	0.029	0.003	−0.012	0.018	0.667	0.042	0.007	−0.009	0.023	0.408	0.03	0.005	−0.011	0.021	0.564	0.042
**Q10**	−0.004	−0.012	0.005	0.410	0.019	−0.004	−0.012	0.005	0.383	0.022	−0.003	−0.012	0.006	0.557	0.018	−0.003	−0.012	0.006	0.480	0.021
**Uric Acid**	−0.005	−0.01	0.00	0.052	0.26	−0.005	−0.01	0.000	0.045	0.264	−0.001	−0.007	0.004	0.619	0.247	−0.002	−0.007	0.004	0.520	0.251
**TRAP**	0.00	−0.002	0.003	0.825	0.187	0.000	−0.002	0.003	0.845	0.188	0.000	−0.002	0.003	0.776	0.187	0.00	−0.002	0.003	0.822	0.188
**FRAP TE**	−0.002	−0.005	0.001	0.133	0.319	−0.002	−0.005	0.001	0.133	0.319	−0.001	−0.004	0.002	0.477	0.313	−0.001	−0.004	0.002	0.473	0.313
**FRAP FE**	−0.002	−0.005	0.001	0.130	0.237	−0.002	−0.005	0.001	0.140	0.238	−0.002	−0.004	0.001	0.268	0.233	−0.002	−0.004	0.001	0.304	0.233
**FRAP WO UA TE**	−0.002	−0.005	0.001	0.287	0.322	−0.002	−0.005	0.002	0.305	0.323	−0.001	−0.005	0.002	0.452	0.32	−0.001	−0.005	0.002	0.507	0.321
**FRAP WO UA FE**	−0.002	−0.004	0.001	0.316	0.172	−0.001	−0.004	0.002	0.354	0.178	−0.002	−0.005	0.001	0.216	0.174	−0.002	−0.005	0.001	0.291	0.179
**TEAC-ABTS**	−0.001	−0.006	0.004	0.597	0.019	−0.001	−0.006	0.004	0.589	0.019	−0.002	−0.007	0.004	0.550	0.019	−0.002	−0.007	0.004	0.528	0.019
**Total Polyphenols**	−0.002	−0.003	0.00	0.063	0.022	−0.002	−0.003	0.000	0.042	0.051	−0.002	−0.004	0.000	0.055	0.023	−0.002	−0.004	0.000	0.020	0.057
**ORAC WO Proteins**	−0.005	−0.01	0.00	0.049	0.144	−0.005	−0.01	0.000	0.050	0.144	−0.005	−0.011	0.00	0.072	0.142	−0.005	−0.011	0.000	0.072	0.142
**ORAC**	0.001	−0.003	0.005	0.513	0.117	0.001	−0.003	0.005	0.496	0.118	0.000	−0.004	0.004	0.922	0.116	0.00	−0.004	0.004	0.871	0.116
**CRP**	−0.019	−0.036	−0.002	0.032	0.152	−0.02	−0.037	−0.003	0.022	0.172	−0.018	−0.036	0.00	0.051	0.149	−0.021	−0.039	−0.003	0.021	0.172
**Adiponectin**	0.012	−0.004	0.029	0.143	0.056	0.014	−0.003	0.03	0.105	0.081	0.01	−0.007	0.028	0.255	0.052	0.014	−0.004	0.032	0.131	0.079
**PAI-I**	−0.007	−0.016	0.002	0.132	0.126	−0.007	−0.016	0.002	0.129	0.126	−0.007	−0.017	0.002	0.141	0.125	−0.008	−0.017	0.002	0.131	0.126
**Resistin**	−0.001	−0.007	0.006	0.870	0.082	−0.001	−0.008	0.006	0.784	0.096	−0.005	−0.013	0.002	0.147	0.092	−0.007	−0.014	0.001	0.081	0.109
**TNF-alfa**	−0.005	−0.014	0.003	0.237	0.082	−0.005	−0.014	0.004	0.260	0.085	−0.005	−0.014	0.004	0.264	0.082	−0.005	−0.014	0.005	0.320	0.084
**IL8**	−0.008	−0.024	0.008	0.328	0.103	−0.008	−0.024	0.008	0.319	0.104	−0.006	−0.023	0.011	0.498	0.101	−0.006	−0.024	0.011	0.465	0.102
**IL6**	0.011	−0.016	0.037	0.441	0.19	0.01	−0.017	0.037	0.455	0.19	0.012	−0.017	0.041	0.408	0.19	0.012	−0.018	0.041	0.437	0.19

Biomarkers: CRP: C-reactive protein; PAI-I: Plasminogen activator; TNF-α: tumor necrosis factor; IL = Interleukin; TRAP: total radical-trapping antioxidant parameter; FRAP: ferric-reducing antioxidant power; TEAC-ABTS: trolox equivalent antioxidant capacity—Azino Bis Thiazoline Sulfonic; ORAC: oxygen radical absorbance capacity; TE: Trolox equivalents; FE: iron equivalents. Abbreviations: WO = without; UA = uric acid; OS = Oxidative Stress. Units: µmol/L for all vitamins, µmol TE/L or FE/L for all TAC assays, mg/dL for Uric acid, mg/L for CRP, pg/mL for all inflammation markers, mg GAE/L (gallic acid equivalents) for total polyphenols. Model 1: adjusted for age (continuous), sex and center. Model 2: additionally, adjusted for energy intake in kcal (continuous). All biomarkers (dependent variables) were log-transformed to approximate a normal distribution. The coefficients β, the corresponding 95% confidence intervals (CI) and R2 are shown (proportion of the variance explained by the independent variables). *p*-values threshold after multiple testing correction = 0.002. The largest β increments as well as the R2 that explain the highest and lowest variance are latticed. The coloured rows in grey show the models with the strongest associations (positive and negative).

**Table 5:** Multivariate linear regression analysis between adherence to the Dietary versions of the Oxidative Balance Score (Nutrient-OBS and Food-OBS) and the biomarkers of nutrient antioxidants (imputed data), OS and inflammation, in the EPIC Granada-Gipuzkoa subsample (N = 210). Imputed data.

	Nutrient-OBS										Food-OBS									
	Model 2					Model 3					Model 2					Model 3				
	β	95% CI		*p*-Value	R^2^	β	95% CI		*p*-Value	R^2^	β	95% CI		*p*-Value	R^2^	β	95% CI		*p*-Value	R^2^
**β-carotene**	0.018	0.00	0.037	0.052	0.073	0.016	−0.003	0.034	0.100	0.13	0.008	−0.012	0.028	0.424	0.059	0.002	−0.019	0.022	0.873	0.118
**Retinol**	−0.003	−0.009	0.004	0.396	0.079	−0.003	−0.01	0.003	0.294	0.133	−0.004	−0.011	0.003	0.288	0.081	−0.005	−0.012	0.002	0.132	0.138
**Tocopherol**	−0.001	−0.011	0.008	0.773	0.015	−0.002	−0.011	0.008	0.697	0.06	0.002	−0.008	0.012	0.658	0.016	0	−0.01	0.011	0.932	0.059
**Ascorbic acid (AA)**	0.012	0.00	0.025	0.054	0.27	0.013	0.00	0.026	0.052	0.297	0.016	0.003	0.03	0.021	0.276	0.017	0.003	0.031	0.018	0.304
**Dehydro AA**	−0.019	−0.122	0.084	0.722	0.339	−0.003	−0.109	0.103	0.950	0.358	0.027	−0.085	0.14	0.632	0.34	0.05	−0.065	0.165	0.393	0.361
**Total Vitamin C**	0.012	−0.002	0.026	0.105	0.311	0.013	−0.002	0.027	0.091	0.34	0.016	0.00	0.031	0.047	0.315	0.017	0.001	0.033	0.038	0.345
**Q9**	0.008	−0.01	0.026	0.372	0.045	0.008	−0.01	0.027	0.382	0.066	0.011	−0.008	0.031	0.267	0.047	0.009	−0.011	0.029	0.402	0.066
**Q10**	−0.003	−0.013	0.007	0.551	0.02	−0.003	−0.013	0.007	0.543	0.089	−0.002	−0.013	0.009	0.708	0.019	−0.004	−0.015	0.006	0.425	0.091
**Uric Acid**	−0.003	−0.009	0.003	0.274	0.254	−0.002	−0.008	0.004	0.557	0.344	0.002	−0.005	0.009	0.542	0.251	0.002	−0.004	0.008	0.531	0.344
**TRAP**	0.001	−0.002	0.003	0.574	0.189	0.001	−0.002	0.004	0.457	0.207	0.001	−0.002	0.004	0.528	0.189	0.001	−0.002	0.004	0.625	0.206
**FRAP TE**	−0.001	−0.004	0.003	0.700	0.312	0.00	−0.004	0.003	0.938	0.362	0.001	−0.002	0.005	0.514	0.313	0.001	−0.003	0.004	0.714	0.363
**FRAP FE**	−0.001	−0.004	0.002	0.443	0.232	−0.001	−0.004	0.002	0.661	0.281	0.00	−0.004	0.003	0.866	0.23	−0.001	−0.004	0.003	0.644	0.281
**FRAP WO UA TE**	0.00	−0.004	0.004	0.921	0.319	0.00	−0.004	0.004	0.953	0.343	0.001	−0.003	0.005	0.677	0.32	0	−0.004	0.005	0.893	0.343
**FRAP WO UA FE**	−0.001	−0.004	0.003	0.587	0.176	−0.001	−0.004	0.003	0.727	0.209	−0.001	−0.005	0.003	0.508	0.176	−0.002	−0.006	0.002	0.329	0.212
**TEAC-ABTS**	0.001	−0.005	0.007	0.745	0.018	0.001	−0.005	0.007	0.757	0.057	0.001	−0.006	0.007	0.768	0.018	0.001	−0.005	0.008	0.727	0.057
**Total Polyphenols**	−0.002	−0.004	0	0.053	0.049	−0.002	−0.004	0	0.046	0.15	−0.003	−0.005	0	0.022	0.056	−0.003	−0.005	−0.001	0.005	0.167
**ORAC WO Proteins**	−0.004	−0.01	0.003	0.249	0.134	−0.004	−0.01	0.003	0.263	0.169	−0.003	−0.01	0.004	0.374	0.131	−0.003	−0.009	0.004	0.447	0.166
**ORAC**	0.002	−0.002	0.007	0.290	0.121	0.003	−0.002	0.008	0.221	0.148	0.001	−0.004	0.006	0.639	0.117	0.001	−0.004	0.006	0.659	0.142
**CRP**	−0.003	−0.023	0.017	0.773	0.151	−0.003	−0.022	0.016	0.759	0.283	−0.001	−0.023	0.021	0.906	0.15	−0.004	−0.025	0.017	0.680	0.283
**Adiponectin**	0.01	−0.01	0.029	0.336	0.073	0.009	−0.011	0.029	0.377	0.101	0.008	−0.013	0.03	0.444	0.072	0.011	−0.011	0.032	0.346	0.102
**PAI-I**	0	−0.011	0.011	0.953	0.116	0.003	−0.008	0.014	0.638	0.182	0.001	−0.011	0.013	0.834	0.116	0.002	−0.01	0.014	0.788	0.181
**Resistin**	0.001	−0.007	0.009	0.861	0.095	0.001	−0.008	0.009	0.890	0.107	−0.007	−0.016	0.002	0.123	0.106	−0.008	−0.017	0.002	0.107	0.119
**TNF-alfa**	−0.004	−0.014	0.006	0.462	0.082	−0.004	−0.015	0.006	0.417	0.127	−0.003	−0.014	0.008	0.589	0.081	−0.005	−0.016	0.007	0.432	0.127
**IL8**	−0.009	−0.028	0.01	0.337	0.103	−0.011	−0.03	0.009	0.278	0.13	−0.007	−0.028	0.014	0.523	0.101	−0.008	−0.03	0.013	0.446	0.127
**IL6**	0.02	−0.012	0.052	0.218	0.194	0.022	−0.01	0.054	0.175	0.259	0.023	−0.011	0.058	0.190	0.195	0.024	−0.011	0.059	0.174	0.259

Biomarkers: CRP: C-reactive protein; PAI-I: Plasminogen activator; TNF-α: tumor necrosis factor; IL = Interleukin; TRAP: total radical-trapping antioxidant parameter; FRAP: ferric-reducing antioxidant power; TEAC-ABTS: trolox equivalent antioxidant capacity—Azino Bis Thiazoline Sulfonic; ORAC: oxygen radical absorbance capacity; TE: Trolox equivalents; FE: iron equivalents. Abbreviations: WO = without; UA = uric acid; OS = oxidative stress. nits: µmol/L for all vitamins, µmol TE/L or FE/L for all TAC assays, mg/dL for Uric acid, mg/L for CRP, pg/mL for all inflammation markers, mg GAE/L (gallic acid equivalents) for total polyphenols. Model 2: adjusted for age (continuous), sex and center, and additionally adjusted for energy intake in kcal (continuous). Model 3: additionally, adjusted for BMI in kg/m^2^ (continuous), smoking status (never, former, and current smoker), and physical activity (inactive, moderately inactive, moderately active and active). All biomarkers (dependent variables) were log-transformed to approximate a normal distribution. The coefficients β, the corresponding 95% confidence intervals (CI) and R2 are shown (proportion of the variance explained by the independent variables). *p*-values threshold after multiple testing correction = 0.002. The largest β increments as well as the R2 that explain the highest and lowest variance are latticed. The coloured rows in grey show the models with the strongest associations (positive and negative).

## Data Availability

The data of this study is preserved by the EPIC-Spain research group. Data are subject to data sharing agreements and are not publicly available.

## Supplementary Materials

The following supporting information can be downloaded at: https://www.mdpi.com/article/10.3390/antiox11020300/s1, Table S1: Summary table of type of components considered in the different OBSs; Table S2: Components of the Food-based-OBS and rationale for their inclusion; Table S3: Characteristics of the components included in the Nutrient-Lifestyle Oxidative Balance Score (NutrientL-OBS) in the EPIC Granada-Gipuzkoa study (N = 14,756) by NutrientL-OBS adherence tertiles; Table S4: Description of components included in the FoodL-OBS in the EPIC Granada-Gipuzkoa study (N = 14,756) by sex (5517 men and 9239 women) and centre (6625 Granada and 8131 Gipuzkoa); Table S5: Dietary intakes of the nutrient components included in the Nutrient- Lifestyle Oxidative Balance Score (NutrientL-OBS) in the EPIC Granada-Gipuzkoa cohort (N = 14,756) by NutrientL-OBS adherence tertiles; Table S6: Dietary intakes of the food components included in the Food-Lifestyle Oxidative Balance Score (FoodL-OBS) in the EPIC Granada-Gipuzkoa cohort (N = 14,756) by FoodL-OBS adherence tertiles; Table S7: Multivariate linear regression analysis between adherence to the Lifestyle Oxidative Balance Score (L-OBS) and the Mediterranean Diet scores (MD) in the Granada-Gipuzkoa EPIC cohort (N = 14,756); Table S8: Multivariate linear regression analysis between adherence to the Dietary Oxidative Balance Score (Nutrient-OBS and Food-OBS) and the Mediterranean Diet scores (MD) in the Granada-Gipuzkoa EPIC cohort (N = 14,756); Table S9: Multivariate linear regression analysis between adherence to the Lifestyle Oxidative Balance Score (L-OBS) and the biomarkers of nutrient antioxidants (imputed data), OS and inflammation, in the EPIC Granada-Gipuzkoa subsample (N = 210); Figure S1: Correlation matrix between the Nutrient, Lifestyle and Food Oxidative Balance Score (NitrientL-OBS, Nutrient-OBS, L-OBS, FoodL-OBS and Food-OBS) and the biomarkers of nutrient antioxidants (imputed data), OS and inflammation, in the EPIC Granada-Gipuzkoa subsample (N = 210).
