# Supplementary material for: Oxidative Balance Scores (OBSs) Integrating Nutrient, Food and Lifestyle Dimensions: Development of the NutrientL-OBS and FoodL-OBS
Source: Antioxidants (Basel). 2022 Jan 31;11(2):300. doi: 10.3390/antiox11020300 (PMC8868253; doi:10.3390/antiox11020300)
Supplement: Supplementary file 1 [file antioxidants-11-00300-s001.zip › antioxidants-1534458-supplementary.pdf]

Supplementary tables and figure

Supplementary Table S1. Summary table of type of components considered in the differents OBSs 2

Supplementary Table S2. Components of the Food-based-OBS and rationale for their inclusion 3

Supplementary Table S3. Characteristics of the components included in the Nutrient-Lifestyle Oxidative Balance Score (NutrientL-OBS) in the EPIC Granada-Gipuzkoa cohort (N=14,756) by NutrientL-OBS adherence tertiles 8

Supplementary Table S4. Description of components included in the FoodL-OBS in the EPIC Granada-Gipuzkoa cohort (N=14,756) by sex (5,517 men and 9,239 women) and centre (6,625 Granada and 8,131 Gipuzkoa) 10

Supplementary Table S5. Dietary intakes of the nutrient components included in the Nutrient-Lifestyle Oxidative Balance Score (NutrientL-OBS) in the EPIC Granada-Gipuzkoa cohort (N=14,756) by NutrientL-OBS adherence tertiles 13

Supplementary Table S6. Dietary intakes of the food components included in the Food-Lifestyle Oxidative Balance Score (FoodL-OBS) in the EPIC Granada-Gipuzkoa cohort (N=14,756) by FoodL-OBS adherence tertiles 14

Supplementary Table S7. Multivariate linear regression analysis between adherence to the Lifestyle Oxidative Balance Score (L-OBS) and the Mediterranean Diet scores (MD) in the Granada-Gipuzkoa EPIC cohort (N=14,756) 15

Supplementary Table S8. Multivariate linear regression analysis between adherence to the Dietary Oxidative Balance Score (Nutrient-OBS and Food-OBS) and the Mediterranean Diet scores (MD) in the Granada-Gipuzkoa EPIC cohort (N=14,756).. 16

Supplementary Table S9. Multivariate linear regression analysis between adherence to the Lifestyle Oxidative Balance Score (L-OBS) and the biomarkers of nutrient antioxidants (imputed data), OS and inflammation, in the EPIC Granada-Gipuzkoa subsample (N=210)...................... 18

Supplementary Figure S1. Correlation matrix between the Nutrient-Lifestyle and Food OxidativeBalance Score (NutrientL-OBS, Nutrient-OBS, L-OBS, FoodL-OBS, and Food-OBS) and the biomarkers of nutrient antioxidants (imputed data), OS and inflammation, in the EPIC Granada-Gipuzkoa subsample (N=210) 19

Supplementary Table S1. Summary table of type of components considered in the differents OBSs

	Range points	Nutrient components (8 components)	Lifestyle components (6 components)	Food-based components (11 components)
**NutrientL-OBS**	*14-70*	*x*	*x*	
**Nutrient-OBS**	*8-40*	*x*		
**L-OBS**	*6-30*		*x*	
**FoodL-OBS**	*14-70*		*x*	*x*
**Food-OBS**	*8-40*			*x*

Supplementary Table S2. Components of the Food-based-OBS and rationale for their inclusion

Food - OBSs components	Foods included in each component	Justification for the inclusion of food OBS component
**Antioxidant foods**		
Vegetable	Vegetables (leafy vegetables, fruiting vegetables, root vegetables, cabbages, mushrooms, grain and pod vegetables, onion, garlic, stalk vegetables, sprouts, mixed salad, and mixed vegetables)	Increased activity of enzymes related to the detoxification of carcinogens (Phase II) or the transformation of procarcinogens into carcinogens (Phase I).<br>Vegetables are one of the food groups with the highest TAC content. Spinach shows the highest antioxidant capacity in the TEAC and FRAP assays followed by peppers, whereas asparagus had the greatest antioxidant capacity in the TRAP assay [1,2].<br>These foods are rich in antioxidant nutrients and bioactive compounds (e.g., β-carotene, folacin, magnesium, calcium, glucosinolates, isothiocyanates, lutein, and indole), all of which play a key role in the antioxidant defense system.
Fruits & juices	Fruits (citrus fruits, apple, pear, grape, stone fruits, berries, banana, kiwi, non-citrus fruits, and other fruits)<br>Fruits and vegetables juices (mixed, citrus, and vegetables juices)	Fruits are one of the food groups with the highest TAC content. The highest antioxidant activities have been reported for berries (i.e., blackberry, redcurrant, and raspberry) regardless of the assay used [1,3].<br>These foods are rich in antioxidant nutrients and bioactive compounds (e.g., β-carotene and α-carotene, and polyphenols), all of which play a key role in the antioxidant defense system.
Legumes	Legumes	Legumes contribute significantly to the TAC. Bean and lentil show the highest antioxidant capacity [4]. These foods contain folacin, iron, isoflavones, protein, vitamin B_6, and fiber, which

		is associated with beneficial alterations to the gut microbiota.
Olive oil	Olive oil	Among oils, extra virgin olive oil had the second-highest antioxidant capacity [1].<br>The bioactive properties of phenolic compounds have been linked to their capacity to inhibit oxidation processes. Several studies have shown that virgin olive oil phenolics protect against OS [5,6]. Hydroxytyrosol has as well-known antioxidant activity as a radical scavenger [7].
Fatty fish	Fatty and very fatty fish	Fatty fish is rich in omega-3 fatty acids, which are beneficial to the human body. These fatty acids compete with pro-inflammatory omega-6 fatty acids. A positive correlation has been established between the amount of fatty fish consumed and the plasma levels of lipoproteins [8]. Meeting requirements of omega-3 fatty acids intake are known to minimize oxidation [9]. Several studies support that food sources of omega-3 fatty acids exhibit high antioxidant capacities [7].
Coffee & tea	Coffee, tea, and herbal teas (coffee, coffee decaffeinated, coffee caffeinated, coffee partially caffeinated, tea, herbal tea, chicory, and substitutes)	These beverages feature a very high TAC content. Among them, coffee had the greatest TAC, regardless of the method of preparation or analysis [1]. Tea contains flavonoids and antioxidants (e.g., epicatechin and quercetin); coffee contains phytochemicals and antioxidants.
**Pro-oxidant foods**		
Meat & meat products	Meat and meat products (red meat, poultry, game, processed meat, and offals).	A higher intake of red meat seems to exert an increased systemic oxidative damage to lipids and proteins [10].<br>Meat and meat products undergo oxidative changes during storage, processing, digestion, and metabolization, making them a potential source of oxidizing agents. High consumption of

		meat and meat products can promote ROS formation at the gastrointestinal tract [11,12]. The foods are rich in SFA and heme-iron, which are will-established drivers of OS in the body [11,12].
Cookies and pastries	Cakes and biscuits (cakes, biscuits, cakes, sweet pies, pastries, puddings, dry cakes, and biscuits). Dough and pastry (dough, pastry, and bread/pizza dough).	High levels of intracellular glucose after intake of these foods lead to an elevation of the anabolic rate, and the increase in the levels of oxidative metabolites [13]. These foods, commonly classified as ultra-processed foods, are also rich in SFA, as well as in simple sugars. Their consumption causes disturbances in the redox status and intestinal microbiota dysbiosis, which in turn also lead to pro-oxidative processes. Together with the disorders triggered by the high intracellular concentrations of simple sugars, systemic oxidative processes in the body are highly favoured [14,15].
Fats & oils	Oils and fats - except olive oil - (all other vegetable oils, butter, margarine, deep frying fats, marine oils, and other animal fat)	High SFA consumption contributes increase in lipogenesis. Increased triacylglycerol synthesis in the endoplasmic reticulum induces stress associated with the unfolded protein response, which is responsible for activating transcription factors related to OS (nuclear factor-kB, NADPH oxidase, and nitric oxide synthase). This increases ROS concentrations in adipocytes [14,16].
Snacks & sauces	Snacks (snacks and aperitif biscuits). Sauces (dressing sauces, mayonnaises, and similars, dessert sauces). Tomato sauce was not included.	These are commonly ultra-processed foods, which have been linked to excess adiposity, and oxide-reductive alterations derived from alterations in cellular mechanisms [17]. High intake of these foods is a key factor in the development of OS disturbances [14].
Cereals & refined products	Bread, crispbread, rusk and salty biscuits, aperitif biscuits, crackers	These foods are also poor in antioxidant nutrients, but rich in simple carbohydrate, thereby inducing hyperglucemia. In addition,

	these foods are sources of food contaminants such as arylamide, acrolein, and furan, which are all generated during the elaboration process. These compounds have been also related to alterations in the oxidative-reductive balance, leading to OS [14,18,19].

Food groups selected for the Food-OBs were those contributing to dietary TAC in the EPIC Granada-Gipuzkoa cohort by TRAP, FRAP, and TEAC methods. Wholegrain cereals and nuts, or sugar-sweetened beverages as antioxidants or pro-oxidants components, respectively, could not be considered in this score due to their low consumption. For the same reason, intake of nutrient supplements was not accounted for. Alcohol intake was considered as a pro-oxidant lifestyle component, as recommended by the majority of the authors of the OBSs [20]. Mechanisms by which nutrients antioxidant and pro-oxidant factors contribute to the oxidative balance of the body are described in Hernández-Ruiz *et al.* 2019 [18].

Abbreviations. OS: Oxidative Stress; ROS: reactive oxygen species; SFA: saturated fatty acids

References

1. Pellegrini, N.; Serafini, M.; Colombi, B.; Del Rio, D.; Salvatore, S.; Bianchi, M.; Brighenti, F. Total antioxidant capacity of plant foods, beverages and oils consumed in Italy assessed by three different in vitro assays. *J. Nutr.* **2003**, *133*, 2812–2819, doi:10.1002/mnfr.200600067.

2. González-Romero, J.; Arranz-Arranz, S.; Verardo, V.; García-Villanova, B.; Guerra-Hernández, E.J. Bioactive compounds and antioxidant capacity of moringa leaves grown in Spain versus 28 leaves commonly consumed in pre-packaged salads. *Processes* **2020**, *8*, 1–20, doi:10.3390/pr8101297.

3. Ruiz-Torralba, A.; Guerra-Hernández, E.J.; García-Villanova, B. Antioxidant capacity, polyphenol content and contribution to dietary intake of 52 fruits sold in spain. *CYTA - J. Food* **2018**, *16*, 1131–1138, doi:10.1080/19476337.2018.1517828.

4. Pellegrini, N.; Serafini, M.; Salvatore, S.; Del Rio, D.; Bianchi, M.; Brighenti, F. Total antioxidant capacity of spices, dried fruits, nuts, pulses, cereals and sweets consumed in Italy assessed by three different in vitro assays. *Mol. Nutr. Food Res.* **2006**, *50*, 1030–1038, doi:10.1002/mnfr.200600067.

5. Quintero-Flórez, A.; Pereira-Caro, G.; Sánchez-Quezada, C.; Moreno-Rojas, J.M.; Gaforio, J.J.; Jimenez, A.; Beltrán, G. Effect of olive cultivar on bioaccessibility and antioxidant activity of phenolic fraction of virgin olive oil. *Eur. J. Nutr.* **2018**, *57*, 1925–1946, doi:10.1007/s00394-017-1475-2.

6. Kouka, P.; Chatzieffraimidi, G.-A.; Raftis, G.; Stagos, D.; Angelis, A.; Stathopoulos, P.; Xynos, N.; Skaltsounis, A.-L.; Tsatsakis, A.M.; Kouretas, D. Antioxidant effects of an olive oil total polyphenolic fraction from a Greek Olea europaea variety in different cell cultures. *Phytomedicine* **2018**, *47*, 135–142, doi:10.1016/j.phymed.2018.04.054.

7. Ricelli, A.; Gionfra, F.; Percario, Z.; De Angelis, M.; Primitivo, L.; Bonfantini, V.; Antonioletti, R.; Bullitta, S.M.; Saso, L.; Incerpi, S.; et al. Antioxidant and Biological Activities of Hydroxytyrosol and Homovanillic Alcohol Obtained from Olive Mill Wastewaters of Extra-Virgin Olive Oil Production. *J. Agric. Food Chem.* **2020**, *68*, 15428–

15439, doi:10.1021/acs.jafc.0c05230.

8. Hudthagosol, C.; Haddad, E.; Jongsuwat, R. Antioxidant activity comparison of walnuts and fatty fish. *J. Med. Assoc. Thail.* **2012**, *95*, 179–188.

9. Ulańczyk, Z.; Grabowicz, A.; Cecerska-Heryć, E.; Śleboda-Taront, D.; Krytkowska, E.; Mozolewska-Piotrowska, K.; Safranow, K.; Kawa, M.P.; Dołęgowska, B.; Machalińska, A. Dietary and lifestyle factors modulate the activity of the endogenous antioxidant system in patients with age-related macular degeneration: Correlations with disease severity. *Antioxidants* **2020**, *9*, 1–19, doi:10.3390/antiox9100954.

10. Ahmad, M.I.; Ijaz, M.U.; ul Haq, I.; Li, C. The role of meat protein in generation of oxidative stress and pathophysiology of metabolic syndromes. *Food Sci. Anim. Resour.* **2020**, *40*, 1–10, doi:10.5851/kosfa.2019.e96.

11. Macho-González, A.; Garcimartín, A.; López-Oliva, M.E.; Bastida, S.; Benedí, J.; Ros, G.; Nieto, G.; Sánchez-Muniz, F.J. Can meat and meat-products induce oxidative stress? *Antioxidants* **2020**, *9*, 1–22, doi:10.3390/antiox9070638.

12. Macho-González, A.; Bastida, S.; Garcimartín, A.; López-Oliva, M.E.; González, P.; Benedí, J.; González-Muñoz, M.J.; Sánchez-Muniz, F.J. Functional Meat Products as Oxidative Stress Modulators: A Review. *Adv. Nutr.* **2021**, *12*, 1514–1539, doi:10.1093/advances/nmaa182.

13. Geto, Z.; Molla, M.D.; Challa, F.; Belay, Y.; Getahun, T. Mitochondrial Dynamic Dysfunction as a Main Triggering Factor for Inflammation Associated Chronic Non-Communicable Diseases. *J. Inflamm. Res.* **2020**, *13*, 97–107, doi:10.2147/JIR.S232009.

14. Martínez Leo, E.E.; Peñafiel, A.M.; Hernández Escalante, V.M.; Cabrera Araujo, Z.M. Ultra-processed diet, systemic oxidative stress, and breach of immunologic tolerance. *Nutrition* **2021**, *91–92*, doi:10.1016/j.nut.2021.111419.

15. Juul, F.; Vaidean, G.; Parekh, N. Ultra-processed Foods and Cardiovascular Diseases: Potential Mechanisms of Action. *Adv. Nutr.* **2021**, *12*, 1673–1680, doi:10.1093/advances/nmab049.

16. Kawasaki, N.; Asada, R.; Saito, A.; Kanemoto, S.; Imaizumi, K. Obesity-induced endoplasmic reticulum stress causes chronic inflammation in adipose tissue. *Sci. Rep.* **2012**, *2*, 799, doi:10.1038/srep00799.

17. Prentice, A.M.; Jebb, S.A. Fast foods, energy density and obesity: a possible mechanistic link. *Obes. Rev. an Off. J. Int. Assoc. Study Obes.* **2003**, *4*, 187–194, doi:10.1046/j.1467-789x.2003.00117.x.

18. Ranjbar, M.; Rotondi, M.A.; Ardern, C.I.; Kuk, J.L. Urinary Biomarkers of Polycyclic Aromatic Hydrocarbons Are Associated with Cardiometabolic Health Risk. *PLoS One* **2015**, *10*, e0137536, doi:10.1371/journal.pone.0137536.

19. Farzan, S.F.; Chen, Y.; Trachtman, H.; Trasande, L. Urinary polycyclic aromatic hydrocarbons and measures of oxidative stress, inflammation and renal function in adolescents: NHANES 2003-2008. *Environ. Res.* **2016**, *144*, 149–157, doi:10.1016/j.envres.2015.11.012.

20. Hernández-Ruiz, Á.; García-Villanova, B.; Guerra-Hernández, E.; Amiano, P.; Ruiz-Canela, M.; Molina-Montes, E. A review of a priori defined oxidative balance scores relative to their components and impact on health outcomes. *Nutrients* **2019**, *11*, 1–36, doi:10.3390/NU11040774.

Supplementary Table S3. Characteristics of the components included in the Nutrient-Lifestyle Oxidative Balance Score (NutrientL-OBS) in the EPIC Granada-Gipuzkoa cohort (N=14,756) by NutrientL-OBS adherence tertiles

	Tertile (T1) N=4991		Tertile 2 (T2) N=5255		Tertile 3 (T3) N=4510		
	N	%	N	%	N	%	*p*-value[a]
**Eight Nutrient components of NutrientL-OBS (in quintiles, Q)**							
**Vitamin C, mg/ d** ᵞ							<0.001
Q1 (1 point)	2052	41.1%	796	15.1%	104	2.31%	
Q2 (2 points)	1491	29.9%	1159	22.1%	301	6.67%	
Q3 (3 points)	929	18.6%	1318	25.1%	704	15.6%	
Q4 (4 points)	410	8.21%	1213	23.1%	1328	29.4%	
Q5 (5 points)	109	2.18%	769	14.6%	2073	46.0%	
**β-carotene, μg/ d** ᵞ							<0.001
Q1 (1 point)	2069	41.50%	762	14.5%	121	2.68%	
Q2 (2 points)	1360	27.20%	1179	22.4%	412	9.14%	
Q3 (3 points)	865	17.30%	1297	24.7%	789	17.5%	
Q4 (4 points)	469	9.40%	1153	21.9%	1329	29.5%	
Q5 (5 points)	228	4.57%	864	16.4%	1859	41.2%	
**α-Tocopherol, mg/ d** ᵞ							<0.001
Q1 (1 point)	1943	38.90%	821	15.6%	188	4.17%	
Q2 (2 points)	1120	22.40%	1159	22.1%	672	14.9%	
Q3 (3 points)	765	15.30%	1130	21.5%	1056	23.4%	
Q4 (4 points)	603	12.10%	1069	20.3%	1279	28.4%	
Q5 (5 points)	560	11.20%	1076	20.5%	1315	29.2%	
**TRAP, μmol trolox** ᵞ							<0.001
Q1 (1 point)	2474	49.60%	473	9.00%	5	0.11%	
Q2 (2 points)	1419	28.40%	1261	24.0%	271	6.01%	
Q3 (3 points)	594	11.90%	1382	26.3%	975	21.6%	
Q4 (4 points)	293	5.87%	1103	21.0%	1555	34.5%	
Q5 (5 points)	211	4.23%	1036	19.7%	1704	37.8%	
**FRAP, μmol iron/d** γ							<0.001
Q1 (1 point)	2469	49.50%	476	9.06%	7	0.16%	
Q2 (2 points)	1471	29.50%	1256	23.9%	224	4.97%	
Q3 (3 points)	615	12.30%	1421	27.0%	915	20.3%	
Q4 (4 points)	261	5.23%	1138	21.7%	1552	34.4%	
Q5 (5 points)	175	3.51%	964	18.3%	1812	40.2%	
**PAC score, -28-28** γ							<0.001
Q1 (1 point)	2453	49.10%	606	11.5%	35	0.78%	
Q2 (2 points)	1497	30.00%	1291	24.6%	327	7.25%	
Q3 (3 points)	698	14.00%	1407	26.8%	895	19.8%	
Q4 (4 points)	227	4.55%	1145	21.8%	1471	32.6%	
Q5 (5 points)	116	2.32%	806	15.3%	1782	39.5%	
**PUFA, g/d** ≠							
Q1 (5 point)	692	13.90%	1155	22.0%	1104	24.5%	
Q2 (4 points)	794	15.90%	1105	21.0%	1052	23.3%	
Q3 (3 points)	915	18.30%	1039	19.8%	997	22.1%	
Q4 (2 points)	1084	21.70%	1051	20.0%	816	18.1%	
Q5 (1 point)	1506	30.20%	905	17.2%	541	12.0%	
**Heme-iron, mg/d** ≠							<0.001
Q1 (5 point)	822	16.50%	1173	22.3%	956	21.2%	
Q2 (4 points)	875	17.50%	1147	21.8%	929	20.6%	

Q3 (3 points)	992	19.90%	1024	19.5%	935	20.7%	
Q4 (2 points)	1059	21.20%	992	18.9%	900	20.0%	
Q5 (1 point)	1243	24.90%	919	17.5%	790	17.5%	
**Six Lifestyle factor components of NutrientL-OBS (in categories)**							
**PA, METs/d γ**							<0.001
Inactive (1 points)	2888	57.90%	1926	36.7%	922	20.4%	
Moderately active (3 points)	1934	38.70%	2821	53.7%	2665	59.1%	
Active (5 points)	169	3.39%	508	9.67%	923	20.5%	
**Alcohol, g/d ≠**							<0.001
>75 g/d, M->50 g/d, W (1 point)	80	1.60%	216	4.11%	71	1.57%	
≤75 g/d, M-≤50 g/d, W (2 points)	228	4.57%	434	8.26%	304	6.74%	
≤50 g/d, M-≤25 g/d, W (3 points)	440	8.82%	939	17.9%	962	21.3%	
≤20 g/d, M-≤15 g/d, W (4 points)	547	11.00%	745	14.2%	754	16.7%	
≤10 g/d, M-≤5 g/d, W (5 points)	3696	74.10%	2921	55.6%	2419	53.6%	
**BMI, kg/m² ≠**							<0.001
≥35 kg/m² (1 point)	752	15.10%	249	4.74%	31	0.69%	
<35 kg/m² (2 points)	1841	36.90%	1180	22.5%	397	8.80%	
≤29.9 kg/m² (3 points)	1309	26.20%	1556	29.6%	1276	28.3%	
≤26.9 kg/m² (4 points)	560	11.20%	1009	19.2%	1201	26.6%	
<25 kg/m² (5 points)	529	10.60%	1261	24.0%	1605	35.6%	
**WC, cm ≠**							<0.001
>102 cm/ men; >88 cm/ women (1 point)	3353	67.20%	1900	36.2%	488	10.8%	
<102 cm/ men; <88 cm/women (5 points)	1638	32.80%	3355	63.8%	4022	89.2%	
**Smoking status (categories) ≠**							<0.001
Never (5 points)	871	17.50%	1443	27.50%	1312	29.10%	
Former (3 points)	551	11.00%	984	18.70%	1086	24.10%	
Current (1 point)	3569	71.50%	2828	53.80%	2112	46.80%	
**Excess energy intake according to total energy expenditure, kcal (categories) ≠**							<0.001
Excess energy intake >30% (1 point)	135	2.70%	230	4.38%	253	5.61%	
Excess energy intake <30% (2 points)	115	2.30%	227	4.32%	267	5.92%	
Excess energy intake <20% (3 points)	180	3.61%	356	6.77%	417	9.25%	
Excess energy intake <10% (4 points)	283	5.67%	559	10.60%	662	14.70%	
Similar energy intake (5 points)	4278	85.70%	3883	73.90%	2911	64.50%	

The age was -years- (mean/SD): T1 (50.9/8.39), T2 (49.5/8.14) and T3 (48.6/7.66); P-value <0.001. The energy intake -kcal/d- (mean/SD): T1 (1798/584), T2 (2111/641) and T3 (2278/612); P-value<0.001. The score NutrientL-OBS (distribution by tertiles, continuous, mean points/SD): T1 (38.7/3.46), T2 (47/1.98), and T3 (54.5/2.97); P-value <0.001

The scores assigned in each quintile or category are indicated in parentheses.

γ Antioxidant components. ≠ Prooxidant components.

[a]Differences between groups have been evaluated using the Chi-square test (categorical variables).

Abbreviations: TRAP: Total Radical Trapping Antioxidant Parameter, FRAP: Ferric Reducing Antioxidant Power PAC Score: Polyphenol An-tioxidant Content Score; BMI: Body Mass Index, WC: Waist Circumference, PA: Physical Activity, PUFA: Polyunsaturated fatty acids; M: Men, W: Women, Q: Quintiles, T=Tertiles

Supplementary Table S4. Description of components included in the FoodL-OBS in the EPIC Granada-Gipuzkoa cohort (N=14,756) by sex (5,517 men and 9,239 women) and centre (6,625 Granada and 8,131 Gipuzkoa)

	Men		Women		P-value[a]	Granada		Gipuzkoa		
	**N**	**%**	**N**	**%**		**N**	**%**	**N**	**%**	*p*-value[a]
**Eleven Food components of FoodL-OBS (in quintiles, Q)**										
**Vegetable γ, g/d**					<0.001					0.073
Q1 (1 point)	1035	18.80%	1917	20.70%		1378	20.80%	1574	19.40%	
Q2 (2 points)	1011	18.30%	1940	21.00%		1335	20.20%	1616	19.90%	
Q3 (3 points)	1084	19.60%	1867	20.20%		1289	19.50%	1662	20.40%	
Q4 (4 points)	1132	20.50%	1819	19.70%		1341	20.20%	1610	19.80%	
Q5 (5 points)	1255	22.70%	1696	18.40%		1282	19.40%	1669	20.50%	
**Fruits and juices γ g/d**					<0.001					<0.001
Q1 (1 point)	1147	20.80%	1805	19.50%		1214	18.30%	1738	21.40%	
Q2 (2 points)	1033	18.70%	1972	21.30%		1616	24.40%	1389	17.10%	
Q3 (3 points)	1007	18.30%	1890	20.50%		1438	21.70%	1459	17.90%	
Q4 (4 points)	1121	20.30%	1830	19.80%		1299	19.60%	1652	20.30%	
Q5 (5 points)	1209	21.90%	1742	18.90%		1058	16.00%	1893	23.30%	
**Legumes γ, g/d**					<0.001					<0.001
Q1 (0.5 points)	599	10.90%	2354	25.50%		1640	24.80%	1313	16.10%	
Q2 (1 point)	672	12.20%	2296	24.90%		1618	24.40%	1350	16.60%	
Q3 (1.5 points)	897	16.30%	2103	22.80%		1622	24.50%	1378	16.90%	
Q4 (2 points)	1311	23.80%	1580	17.10%		1104	16.70%	1787	22.00%	
Q5 (2.5 points)	2038	36.90%	906	9.81%		641	9.68%	2303	28.30%	
**Olive oil γ, g/d**					<0.001					<0.001
Q1 (0.5 points)	1469	26.60%	1483	16.10%		426	6.43%	2526	31.10%	
Q2 (1 point)	663	12.00%	2288	24.80%		1755	26.50%	1196	14.70%	
Q3 (1.5 points)	705	12.80%	2246	24.30%		1822	27.50%	1129	13.90%	
Q4 (2 points)	991	18.00%	1960	21.20%		1487	22.40%	1464	18.00%	
Q5 (2.5 points)	1689	30.60%	1262	13.70%		1135	17.10%	1816	22.30%	
**Fatty fish γ, g/d**					<0.001					<0.001
Q1 (0.5 points)	660	12.00%	2301	24.90%		1969	29.70%	992	12.20%	
Q2 (1 point)	745	13.50%	2197	23.80%		1576	23.80%	1366	16.80%	
Q3 (1.5 points)	981	17.80%	1970	21.30%		1250	18.90%	1701	20.90%	
Q4 (2 points)	1301	23.60%	1650	17.90%		1081	16.30%	1870	23.00%	
Q5 (2.5 points)	1830	33.20%	1121	12.10%		749	11.30%	2202	27.10%	
**Coffee and tea γ, g/d**					<0.001					<0.001
Q1 (1 point)	1052	19.10%	1900	20.60%		1938	29.30%	1014	12.50%	
Q2 (2 points)	1437	26.00%	1518	16.40%		1270	19.20%	1685	20.70%	
Q3 (3 points)	1352	24.50%	1912	20.70%		1390	21.00%	1874	23.00%	
Q4 (4 points)	923	16.70%	1715	18.60%		1046	15.80%	1592	19.60%	
Q5 (5 points)	753	13.60%	2194	23.70%		981	14.80%	1966	24.20%	
**Meat and meat products *, g/d**					<0.001					<0.001
Q5 (1 points)	2184	39.6%	767	8.3%		617	9.3%	2334	28.7%	
Q4 (2 points)	1394	25.3%	1557	16.8%		904	13.6%	2047	25.2%	
Q3 (3 points)	903	16.4%	2048	22.2%		1273	19.2%	1678	20.6%	
Q2 (4 points)	596	10.8%	2355	25.5%		1754	26.5%	1197	14.7%	
Q1 (5 points)	440	8.0%	2512	27.2%		2077	31.3%	875	10.8%	
**Cookies and pastries *, g/d (tertiles)**					<0.001					<0.001
Q5 (0.5 points)	781	14.2%	1756	19.0%		1281	19.3%	1256	15.4%	
Q4 (1 point)	781	14.2%	1737	18.8%		1302	19.7%	1216	15.0%	
Q3 (1.5 points)	939	17.0%	1620	17.5%		1024	15.5%	1535	18.9%	
Q2 (2 points)	908	16.5%	1630	17.6%		1016	15.3%	1522	18.7%	

Q1 (2.5 points)	2108	38.2%	2496	27.0%		2002	30.2%	2602	32.0%	
**Fats and oils ≠, g/d (median)**					<0.001					<0.001
Q5 (1 point)	1525	27.6%	920	9.96%		205	3.09%	2240	27.5%	
Q4 (2 points)	787	14.3%	1657	17.9%		785	11.8%	1659	20.4%	
Q3 (3 points)	573	10.4%	1871	20.3%		1397	21.1%	1047	12.9%	
Q2 (4 points)	606	11.0%	1839	19.9%		1578	23.8%	867	10.7%	
Q1 (5 points)	2026	36.7%	2952	32.0%		2660	40.2%	2318	28.5%	
**Snacks and sauces ≠, g/d**					<0.001					<0.001
Q5 (0.5 points)	1812	32,8%	1139	12.3%		582	8.78%	2369	29.1%	
Q4 (1 point)	1285	23.3%	1666	18.0%		1067	16.1%	1884	23.2%	
Q3 (1.5 points)	1010	18.3%	1913	20.7%		1281	19.3%	1642	20.2%	
Q2 (2 points)	690	12.5%	2287	24.8%		1699	25.6%	1278	15.7%	
Q1 (2.5 points)	720	13.1%	2234	24.2%		1966	30.1%	958	11.8%	
**Cereals and refined products≠, g/d (quintiles)**					<0.001					<0.001
Q5 (0.5 points)	2012	36.5%	939	10.2%		863	13.0%	2088	25.7%	
Q4 (1 point)	1374	24.9%	1576	17.1%		1188	17.9%	1762	21.7%	
Q3 (1.5 points)	909	16.5%	1958	21.2%		1373	20.7%	1494	18.4%	
Q2 (2 points)	718	13.0%	2317	25.1%		1504	22.7%	1531	18.8%	
Q1 (2.5 points)	504	9.14%	2449	26.5%		1697	25.6%	1256	15.4%	
**Six Lifestyle factors components of FoodL-OBS**										
**PA, METs/ d (categories) γ**					<0.001					<0.001
Inactive (1 point))	1132	20.50%	4604	49.80%		3486	52.60%	2250	27.70%	
Moderate (3 points)	3174	57.50%	4246	46.00%		2705	40.80%	4715	58.00%	
Active (5 points)	1211	22.00%	389	4.21%		434	6.55%	1166	14.30%	
**Alcohol consumption, g/ d (categories)≠**					<0.001					<0.001
>75 g/d, M->50 g/d, W (1 point)	338	6.13%	29	0.31%		27	0.41%	340	4.18%	
≤75 g/d, M-≤50 g/d, W (2 points)	641	11.60%	325	3.52%		101	1.52%	865	10.60%	
≤50 g/d, M-≤25 g/d, W (3 points)	1855	33.60%	486	5.26%		516	7.79%	1825	22.40%	
≤20 g/d, M-≤15 g/d, W (4 points)	838	15.20%	1208	13.10%		681	10.30%	1365	16.80%	
≤10 g/d, M-≤5 g/d, W (5 points)	1845	33.40%	7191	77.80%		5300	80.00%	3736	45.90%	
**BMI, kg/m² (categories)≠**					<0.001					<0.001
≥35 kg/m² (1 point)	180	3.26%	852	9.22%		688	10.40%	344	4.23%	
<35 kg/m² (2 points)	1306	23.70%	2112	22.90%		1923	29.00%	1495	18.40%	
≤29.9 kg/m² (3 points)	2006	36.40%	2135	23.10%		1793	27.10%	2348	28.90%	
≤26.9 kg/m² (4 points)	1194	21.60%	1576	17.10%		1004	15.20%	1766	21.70%	
<25 kg/m² (5 points)	831	15.10%	2564	27.80%		1217	18.40%	2178	26.80%	
**Waist circumference, cm (categories)≠**					<0.001					<0.001
>102 cm/ men; >88 cm/ women (1 point)	1814	32.90%	3927	42.50%		3229	48.70%	2512	30.90%	
<102 cm/ men; <88 cm/women (5 points)	3703	67,10%	5312	57,50%		3396	51,30%	5619	69,10%	
**Smoking habit (categories)**					<0,001					<0,001
Current (1 point)	2164	39.20%	1462	15.80%		1206	18.20%	2420	29.80%	
Former (3 points)	1679	30.40%	942	10.20%		996	15.00%	1625	20.00%	
Never (5 points)	1674	30.30%	6835	74.00%		4423	66.80%	4086	50.30%	
**Excess energy intake according to total energy expenditure, kcal (categories)**					<0.001					<0.001
Excess energy intake >30% (1 point)	296	5.37%	322	3.49%		170	2.57%	448	5.51%	
Excess energy intake <30% (2 points)	317	5.75%	292	3.16%		155	2.34%	454	5.58%	
Excess energy intake <20% (3 points)	485	8.79%	468	5.07%		270	4.08%	683	8.40%	
Excess energy intake <10% (4 points)	735	13.30%	769	8.32%		439	6.63%	1065	13.10%	
Similar energy intake (5 points)	3684	66.80%	7388	80.00%		5591	84.40%	5481	67.40%	
**Description of scores and distribution by sex and center FoodL-OBS**										
	**By sex**					**By center**				
**FoodL-OBS (tertiles)**					0.286					<0.001
**T1**	1805	32.70%	3092	33.50%		2500	37.70%	2397	29.50%	

**T2**	2108	38.20%	3410	36.90%		2432	36.70%	3086	38.00%	
**T3**	1604	29.10%	2737	29.60%		1693	25.60%	2648	32.60%	

The score FoodL-OBS was (mean points /SD): men (50.2/6.03), and woman (50.2/6.08), P-value 0.944; in the EPIC- Granada center (50.7/6.03) and in the in the EPIC-Gipuzkoa center (47.7/6.56), P-value <0.001.

The scores assigned in each quintile or category are indicated in parentheses.

[γ] Antioxidant components [≠] Prooxidant components

[a]Differences between groups have been evaluated using the Chi-square test (categorical variables). Descriptives are shown in frequencies and percentages for categorical variables.

Abbreviations: BMI: Body Mass Index, WC: Waist Circumference, PA: Physical Activity, M: Men, W: Women, Q: Quintiles, T=Tertiles

Supplementary Table S5. Dietary intakes of the nutrient components included in the Nutrient-Lifestyle Oxidative Balance Score (NutrientL-OBS) in the EPIC Granada-Gipuzkoa cohort (N=14,756) by NutrientL-OBS adherence tertiles.

	**Tertile (T1)**		**Tertile 2 (T2)**		**Tertile 3 (T3)**		
	**N=4991**		**N=5255**		**N=4510**		
	**Median**	**IQR**	**Median**	**IQR**	**Median**	**IQR**	***p*-value**
**Vitamin C, mg/ d** γ	91.6	66.2-118	133	103-168	188	150;238	<0.001
**β-carotene, μg/ d** γ	1494	1096-2050	2134	1602-2917	3000	2234-4163	<0.001
**α-Tocopherol, mg/ d** γ	9.59	7.12-14.0	11.6	8.86-16.6	13.4	10.5-18.0	<0.001
**TRAP, μmol trolox** γ	1951	1461-2728	3096	2341-4736	4277	3286-6025	<0.001
**FRAP, μmol iron/d** γ	6616	5131-8750	9896	7721-13708	13242	10466-17226	<0.001
**PAC score, -28-28** γ	-10	-16.00-3.00	1	-6.00-8.00	9	2.00-15.0	<0.001
**PUFA, g/d** ≠	10.4	7.44-15.6	11.5	8.24-17.5	11.7	8.63-17.1	<0.001
**Heme-iron, mg/d** ≠	1.41	0.88-2.12	1.46	0.94-2.17	1.32	0.87-1.98	<0.001

γ Antioxidant components ≠ Prooxidant components

The differences between tertiles have been assessed using the Kruskal–Wallis test (continuous variables). Descriptives are shown in medians and interquartile ranges (IQR).

Abbreviations: TRAP: Total Radical Trapping Antioxidant Parameter, FRAP: Ferric Reducing Antioxidant Power PAC Score: Polyphenol Antioxidant Content Score.

Supplementary Table S6. Dietary intakes of the food components included in the Food-Lifestyle Oxidative Balance Score (FoodL-OBS) in the EPIC Granada-Gipuzkoa cohort (N=14,756) by FoodL-OBS adherence tertiles

	Tertile 1 (T1); N=4917		Tertile 2 (T2); N=4900		Tertile 3 (T3); N=4939		
	**Median**	**IQR**	**Median**	**IQR**	**Median**	**IQR**	**P-value**
**Vegetable, g/d** γ	170	111-245	211	147-298	279	201-378	<0.001
**Fruits and juices, g/d** γ	228	117-347	295	187-442	390	254-556	<0.001
**Legumes, g/d** γ·	39.6	22.6-65.7	40.3	24.3-65.0	43.6	25.0-66.7	0.004
**Olive oil, g/d** γ·	10.8	0.00-20.7	18.3	10.4-27.1	23.8	16.3-32.3	<0.001
**Fatty fish, g/d** γ	7.5	0.60-16.3	8.76	2.44-17.9	10.9	3.66-22.4	<0.001
**Coffee and tea, g/d** γ	80.7	8.04-168	102	26.0-201	150	53.6-250	<0.001
**Meat and meat products, g/d** ≠	135	95.0-181	108	73.8-148	91.8	64.2-127	<0.001
**Cookies and pastries, g/d** ≠	27.1	0.00-72.7	21.3	0.00-60.0	13.4	0.00-45.4	<0.001
**Fats and oils, g/d** ≠	12.9	0.93-27.4	5.11	0.00-16.1	1.2	0.00;9.00	<0.001
**Snacks and sauces, g/d** ≠	3.75	0.00-16.3	1.71	0.00-7.80	0.64	0.00-4.71	<0.001
**Cereals and refined products, g/d** ≠	171	120-240	139	93.6-197	112	71.5-161	<0.001

γ Antioxidant components ≠ Prooxidant components
The differences between tertiles have been assessed using the Kruskal–Wallis test (continuous variables). Descriptives are shown in medians and interquartile ranges (IQR).
Abbreviations: T=Tertiles

Supplementary Table S7. Multivariate linear regression analysis between adherence to the Lifestyle Oxidative Balance Score (L-OBS) and the Mediterranean Diet scores (MD) in the Granada-Gipuzkoa EPIC cohort (N=14,756).

	Model 1					Model 2				
	β	95% CI		P-value	R^2	β	95% CI		P-value	R^2
**rMED**	0.001	-0.001	0.003	<0.001	0.088	0.008	0.006	0.01	<0.001	0.156
**aMED**	0.022	0.016	0.029	<0.001	0.008	0.032	0.025	0.039	<0.001	0.016
**MedDietScore2004**	0.001	0.001	0.002	<0.001	0.135	0.005	0.005	0.006	<0.001	0.300
**MedDietScore2005**	0.002	0.001	0.003	<0.001	0.029	0.003	0.003	0.004	<0.001	0.047
**MedDietScore2007**	0.006	0.005	0.006	<0.001	0.056	0.007	0.006	0.007	<0.001	0.060
**ShortMedQ**	0.017	0.015	0.018	<0.001	0.055	0.019	0.017	0.021	<0.001	0.061
**Lbas**	0.004	0.003	0.005	<0.001	0.015	0.006	0.005	0.008	<0.001	0.036
**PREDIMED**	-0.002	-0.003	-0.001	<0.001	0.101	0.001	0.000	0.001	<0.001	0.163
**MDS1995**	-0.009	-0.013	-0.005	<0.001	0.002	-0.002	-0.006	0.002	<0.001	0.016
**MDS2013**	-0.002	-0.005	0.002	<0.001	0.031	0.006	0.002	0.01	<0.001	0.050
**MDSS**	0.003	0.002	0.005	<0.001	0.013	0.007	0.006	0.008	<0.001	0.050
**MSDPS**	0.001	0.000	0.002	<0.001	0.008	0.001	0.000	0.002	<0.001	0.009
**MDQI**	-0.010	-0.012	-0.009	<0.001	0.034	-0.009	-0.011	-0.008	<0.001	0.035
**MDP2003**	0.005	0.004	0.006	<0.001	0.024	0.004	0.003	0.005	<0.001	0.030
**MDPA2002**	-0.002	-0.003	-0.001	<0.001	0.183	0.003	0.002	0.004	<0.001	0.321
**MDP2006**	0.001	0.000	0.001	<0.001	0.086	0.005	0.004	0.005	<0.001	0.269
**MMD2005**	-0.004	-0.008	-0.001	<0.001	0.068	0.007	0.004	0.01	<0.001	0.120
**MEDLIFE**	0.000	-0.001	0.001	<0.001	0.047	0.002	0.001	0.003	<0.001	0.060
**ITAMED**	0.009	0.006	0.013	<0.001	0.016	0.013	0.009	0.016	<0.001	0.021
**MDScale2003**	-0.003	-0.007	0.001	<0.001	0.043	0.002	-0.001	0.006	<0.001	0.053

Mediterranean Diet scores (DM): aMED: the Alternate MD Index; rMED: the Relative MD Score; MedDietScore: MD Score-2004, MD Score-2005, MD Score-207; ShortMedQ: the Cardioprotective MD Score; Lbas: the literature-based adherence score to the MD; PREDIMED: the Mediterranean food pattern of the PREDIMED Study; MDS: the MDS-1995, MDS-2003; MDSS: Mediterranean Diet Serving Score; MSDPS: Mediterranean-Style Dietary Pattern Score; MDQI: the MD Quality Index; MDP: the Mediterranean Dietary Pattern-2002; Mediterranean Dietary Pattern-2006; MMD2005: the Modified MD-2005; MEDLIDE: the Mediterranean Lifestyle Index; ITAMED: Italian Mediterranean Index; MDScale2003: MDScale-2003;
MDQI: scoring goes in the opposite way (high refers to low adherence).

Model 1: adjusted for age (continuous), sex and center.
Model 2: additionally, adjusted for energy intake in kcal (continuous).
All MD score (dependent variables) were log-transformed to approximate a normal distribution. The coefficients β, the corresponding 95% confidence intervals (CI) and R2 are shown (proportion of the variance explained by the independent variables).

Supplementary Table S8. Multivariate linear regression analysis between adherence to the Dietary Oxidative Balance Score (Nutrient-OBS and Food-OBS) and the Mediterranean Diet scores (MD) in the Granada-Gipuzkoa EPIC cohort (N=14,756)

Model 1										
	Nutrient-OBS					Food-OBS				
	β	95% CI		P-value	R^2	β	95% CI		P-value	R^2
**rMED**	0.032	0.031	0.033	<0.001	0.306	0.039	0.038	0.040	<0.001	0.309
**aMED**	0.080	0.076	0.084	<0.001	0.087	0.096	0.091	0.101	<0.001	0.087
**MedDietScore2004**	0.011	0.010	0.011	<0.001	0.301	0.010	0.009	0.010	<0.001	0.225
**MedDietScore2005**	0.008	0.007	0.008	<0.001	0.120	0.011	0.011	0.012	<0.001	0.173
**MedDietScore2007**	0.011	0.011	0.011	<0.001	0.243	0.017	0.017	0.018	<0.001	0.402
**ShortMedQ**	0.027	0.026	0.028	<0.001	0.162	0.043	0.042	0.044	<0.001	0.252
**Lbas**	0.018	0.018	0.019	<0.001	0.172	0.025	0.024	0.025	<0.001	0.208
**PREDIMED**	0.014	0.014	0.015	<0.001	0.332	0.017	0.016	0.017	<0.001	0.312
**MDS1995**	0.048	0.045	0.050	<0.001	0.098	0.05	0.047	0.053	<0.001	0.075
**MDS2013**	0.043	0.040	0.045	<0.001	0.110	0.038	0.035	0.04	<0.001	0.073
**MDSS**	0.015	0.015	0.016	<0.001	0.101	0.019	0.019	0.02	<0.001	0.111
**MSDPS**	0.009	0.008	0.010	<0.001	0.073	0.014	0.013	0.014	<0.001	0.111
**MDQI**	-0.029	-0.03	-0.028	<0.001	0.190	-0.042	-0.043	-0.041	<0.001	0.273
**MDP2003**	0.009	0.009	0.010	<0.001	0.093	0.017	0.016	0.017	<0.001	0.202
**MDPA2002**	0.022	0.021	0.022	<0.001	0.544	0.022	0.021	0.022	<0.001	0.433
**MDP2006**	0.011	0.011	0.012	<0.001	0.274	0.014	0.013	0.014	<0.001	0.277
**MMD2005**	0.043	0.041	0.045	<0.001	0.167	0.043	0.041	0.046	<0.001	0.139
**MEDLIFE**	0.013	0.013	0.014	<0.001	0.182	0.016	0.016	0.017	<0.001	0.185
**ITAMED**	0.036	0.034	0.038	<0.001	0.085	0.052	0.049	0.054	<0.001	0.113
**MDScale2003**	0.044	0.042	0.046	<0.001	0.131	0.052	0.049	0.055	<0.001	0.128
**Model 2**										
	Nutrient-OBS					Food-OBS				
	β	95% CI		P-value	R^2	β	95% CI		P-value	R^2
**rMED**	0.030	0.029	0.031	<0.001	0.323	0.043	0.042	0.044	<0.001	0.408
**aMED**	0.080	0.076	0.085	<0.001	0.087	0.101	0.096	0.106	<0.001	0.101
**MedDietScore2004**	0.009	0.008	0.009	<0.001	0.383	0.011	0.011	0.012	<0.001	0.410
**MedDietScore2005**	0.007	0.007	0.008	<0.001	0.121	0.012	0.012	0.013	<0.001	0.201
**MedDietScore2007**	0.012	0.011	0.012	<0.001	0.254	0.018	0.017	0.018	<0.001	0.413
**ShortMedQ**	0.029	0.027	0.030	<0.001	0.166	0.044	0.043	0.045	<0.001	0.262
**Lbas**	0.018	0.018	0.019	<0.001	0.173	0.026	0.025	0.027	<0.001	0.243
**PREDIMED**	0.013	0.013	0.014	<0.001	0.347	0.018	0.017	0.018	<0.001	0.410
**MDS1995**	0.046	0.044	0.048	<0.001	0.099	0.054	0.051	0.057	<0.001	0.100
**MDS2013**	0.040	0.038	0.042	<0.001	0.114	0.041	0.039	0.044	<0.001	0.100
**MDSS**	0.014	0.013	0.015	<0.001	0.110	0.021	0.020	0.022	<0.001	0.158
**MSDPS**	0.009	0.009	0.010	<0.001	0.075	0.014	0.013	0.015	<0.001	0.115
**MDQI**	-0.032	-0.033	-0.031	<0.001	0.220	-0.042	-0.043	-0.041	<0.001	0.273
**MDP2003**	0.011	0.01	0.011	<0.001	0.126	0.017	0.016	0.017	<0.001	0.204
**MDPA2002**	0.019	0.019	0.020	<0.001	0.589	0.024	0.024	0.024	<0.001	0.624

**MDP2006**	0.009	0.009	0.009	<0.001	0.367	0.016	0.015	0.016	<0.001	0.500
**MMD2005**	0.037	0.035	0.039	<0.001	0.187	0.049	0.046	0.051	<0.001	0.206
**MEDLIFE**	0.013	0.013	0.014	<0.001	0.182	0.017	0.016	0.018	<0.001	0.210
**ITAMED**	0.037	0.035	0.039	<0.001	0.085	0.054	0.051	0.056	<0.001	0.122
**MDScale2003**	0.043	0.041	0.046	<0.001	0.132	0.055	0.052	0.058	<0.001	0.146
**Model 3**										
	**Nutrient-OBS**					**Food-OBS**				
	**β**	**95% CI**		**P-value**	**R^2**	**β**	**95% CI**		**P-value**	**R^2**
**rMED**	0.030	0.029	0.031	<0.001	0.324	0.043	0.041	0.044	<0.001	0.412
**aMED**	0.079	0.075	0.084	<0.001	0.088	0.101	0.095	0.106	<0.001	0.103
**MedDietScore2004**	0.008	0.008	0.009	<0.001	0.387	0.011	0.011	0.012	<0.001	0.416
**MedDietScore2005**	0.007	0.007	0.008	<0.001	0.124	0.012	0.012	0.012	<0.001	0.206
**MedDietScore2007**	0.011	0.011	0.012	<0.001	0.261	0.018	0.017	0.018	<0.001	0.428
**ShortMedQ**	0.028	0.027	0.029	<0.001	0.172	0.044	0.043	0.045	<0.001	0.273
**Lbas**	0.018	0.018	0.019	<0.001	0.177	0.026	0.025	0.027	<0.001	0.249
**PREDIMED**	0.013	0.013	0.014	<0.001	0.348	0.018	0.017	0.018	<0.001	0.411
**MDS1995**	0.046	0.044	0.049	<0.001	0.100	0.054	0.051	0.057	<0.001	0.101
**MDS2013**	0.040	0.038	0.043	<0.001	0.115	0.041	0.038	0.044	<0.001	0.101
**MDSS**	0.014	0.013	0.015	<0.001	0.114	0.021	0.020	0.022	<0.001	0.163
**MSDPS**	0.009	0.009	0.010	<0.001	0.077	0.014	0.013	0.015	<0.001	0.118
**MDQI**	-0.032	-0.033	-0.031	<0.001	0.221	-0.042	-0.043	-0.041	<0.001	0.277
**MDP2003**	0.010	0.010	0.011	<0.001	0.131	0.016	0.016	0.017	<0.001	0.212
**MDPA2002**	0.019	0.019	0.020	<0.001	0.591	0.024	0.023	0.024	<0.001	0.628
**MDP2006**	0.009	0.009	0.009	<0.001	0.370	0.016	0.015	0.016	<0.001	0.505
**MMD2005**	0.037	0.035	0.039	<0.001	0.188	0.048	0.046	0.051	<0.001	0.208
**MEDLIFE**	0.013	0.013	0.014	<0.001	0.182	0.017	0.016	0.018	<0.001	0.211
**ITAMED**	0.037	0.035	0.039	<0.001	0.088	0.053	0.051	0.056	<0.001	0.126
**MDScale2003**	0.044	0.041	0.046	<0.001	0.133	0.055	0.052	0.058	<0.001	0.147

Mediterranean Diet scores (DM): aMED: the Alternate MD Index; rMED: the Relative MD Score; MedDietScore: MD Score-2004, MD Score-2005, MD Score-207; ShortMedQ: the Cardioprotective MD Score; Lbas: the literature-based adherence score to the MD; PREDIMED: the Mediterranean food pattern of the PREDIMED Study; MDS: the MDS-1995, MDS-2003; MDSS: Mediterranean Diet Serving Score; MSDPS: Mediterranean-Style Dietary Pattern Score; MDQI: the MD Quality Index; MDP: the Mediterranean Dietary Pattern-2002; Mediterranean Dietary Pattern-2006; MMD2005: the Modified MD-2005; MEDLIDE: the Mediterranean Lifestyle Index; ITAMED: Italian Mediterranean Index; MDScale2003: MDScale-2003.
MDQI: scoring goes in the opposite way (high refers to low adherence).

Model 1: adjusted for age (continuous), sex and center;
Model 2: additionally, adjusted for energy intake in kcal (continuous);
Model 3: additionally, adjusted for BMI in kg/m² (continuous), smoking status (never, former, and current smoker), and physical activity levels (inactive, moderate and active).
All MD score (dependent variables) were log-transformed to approximate a normal distribution. The coefficients β, the corresponding 95% confidence intervals (CI) and R2 are shown (proportion of the variance explained by the independent variables).

Supplementary Table S9. Multivariate linear regression analysis between adherence to the Lifestyle Oxidative Balance Score (L-OBS) and the biomarkers of nutrient antioxidants (imputed data), OS and inflammation, in the EPIC Granada-Gipuzkoa subsample (N=210).

	Model 1					Model 2				
	**β**	**95% CI**		***p*-value**	**R^2**	**β**	**95% CI**		***p*-value**	**R^2**
**β-carotene**	0.041	-0.101	0.182	0.573	0.051	0.054	-0.089	0.197	0.459	0.058
**Retinol**	-0.05	-0.098	-0.002	0.041	0.094	-0.052	-0.101	-0.004	0.035	0.095
**Tocopherol**	-0.003	-0.074	0.068	0.933	0.010	-0.008	-0.080	0.063	0.817	0.015
**Ascorbic acid**	0.054	-0.043	0.15	0.280	0.259	0.060	-0.038	0.158	0.229	0.262
**Dehydroascorbic acid**	-0.053	-0.843	0.737	0.895	0.338	-0.025	-0.825	0.776	0.952	0.339
**Total Vitamin C**	0.038	-0.071	0.148	0.494	0.299	0.049	-0.062	0.160	0.390	0.304
**Q9**	-0.08	-0.218	0.058	0.258	0.033	-0.100	-0.238	0.039	0.160	0.050
**Q10**	-0.052	-0.128	0.024	0.179	0.025	-0.057	-0.134	0.019	0.142	0.029
**Uric Acid**	-0.056	-0.102	-0.01	0.018	0.267	-0.060	-0.107	-0.014	0.011	0.273
**TRAP**	-0.007	-0.028	0.014	0.507	0.189	-0.008	-0.029	0.013	0.466	0.190
**FRAP TE**	-0.034	-0.059	-0.009	0.009	0.334	-0.035	-0.060	-0.009	0.008	0.334
**FRAP FE**	-0.023	-0.047	0.001	0.059	0.242	-0.023	-0.047	0.002	0.069	0.242
**FRAP WO UA TE**	-0.032	-0.061	-0.002	0.035	0.332	-0.031	-0.061	-0.001	0.042	0.333
**FRAP WO UA FE**	-0.015	-0.042	0.012	0.265	0.173	-0.013	-0.040	0.014	0.348	0.178
**TEAC-ABTS**	-0.036	-0.081	0.009	0.123	0.029	-0.037	-0.083	0.009	0.113	0.030
**Total Polyphenols**	-0.004	-0.019	0.012	0.657	0.006	-0.006	-0.022	0.009	0.422	0.034
**ORAC WO Proteins**	-0.045	-0.091	0.002	0.061	0.143	-0.046	-0.093	0.002	0.060	0.143
**ORAC**	-0.01	-0.044	0.025	0.591	0.117	-0.009	-0.044	0.027	0.631	0.117
**CRP**	-0.321	-0.47	-0.172	<0.001	0.202	-0.352	-0.500	-0.204	<0.001	0.232
**Adiponectin**	0.121	-0.031	0.272	0.120	0.058	0.149	-0.002	0.300	0.054	0.086
**PAI-I**	-0.126	-0.208	-0.045	0.003	0.154	-0.130	-0.213	-0.048	0.002	0.156
**Resistin**	-0.023	-0.086	0.039	0.466	0.085	-0.032	-0.095	0.031	0.318	0.100
**TNF-alfa**	-0.039	-0.118	0.039	0.328	0.080	-0.035	-0.114	0.045	0.392	0.083
**IL8**	0.016	-0.13	0.162	0.832	0.099	0.013	-0.135	0.161	0.865	0.099
**IL6**	-0.075	-0.32	0.169	0.547	0.189	-0.084	-0.332	0.164	0.508	0.190

Biomarkers: CRP: C-reactive protein; PAI-I: Plasminogen activator; TNF-α: tumor necrosis factor; IL=Interleukin; TRAP: total radical-trapping antioxidant parameter; FRAP: ferric-reducing antioxidant power; TEAC-ABTS: trolox equivalent antioxidant capacity—Azino Bis Thiazoline Sulfonic; ORAC: oxygen radical absorbance capacity; TE: Trolox equivalents; FE: iron equivalents. The coloured rows in grey show the models with the strongest associations (positive and negative).

Abbreviations: WO = without; UA = uric acid; OS = oxidative stress

Model 1: adjusted for age (continuous), sex and center.
Model 2: additionally, adjusted for energy intake in Kcal (continuous).
All biomarkers (dependent variables) were log-transformed to approximate a normal distribution. The coefficients β, the corresponding 95% confidence intervals (CI) and R2 are shown (proportion of the variance explained by the independent variables). P-values threshold after multiple testing correction = 0.002. The largest β increments as well as the R2 that explain the highest and lowest variance are latticed.

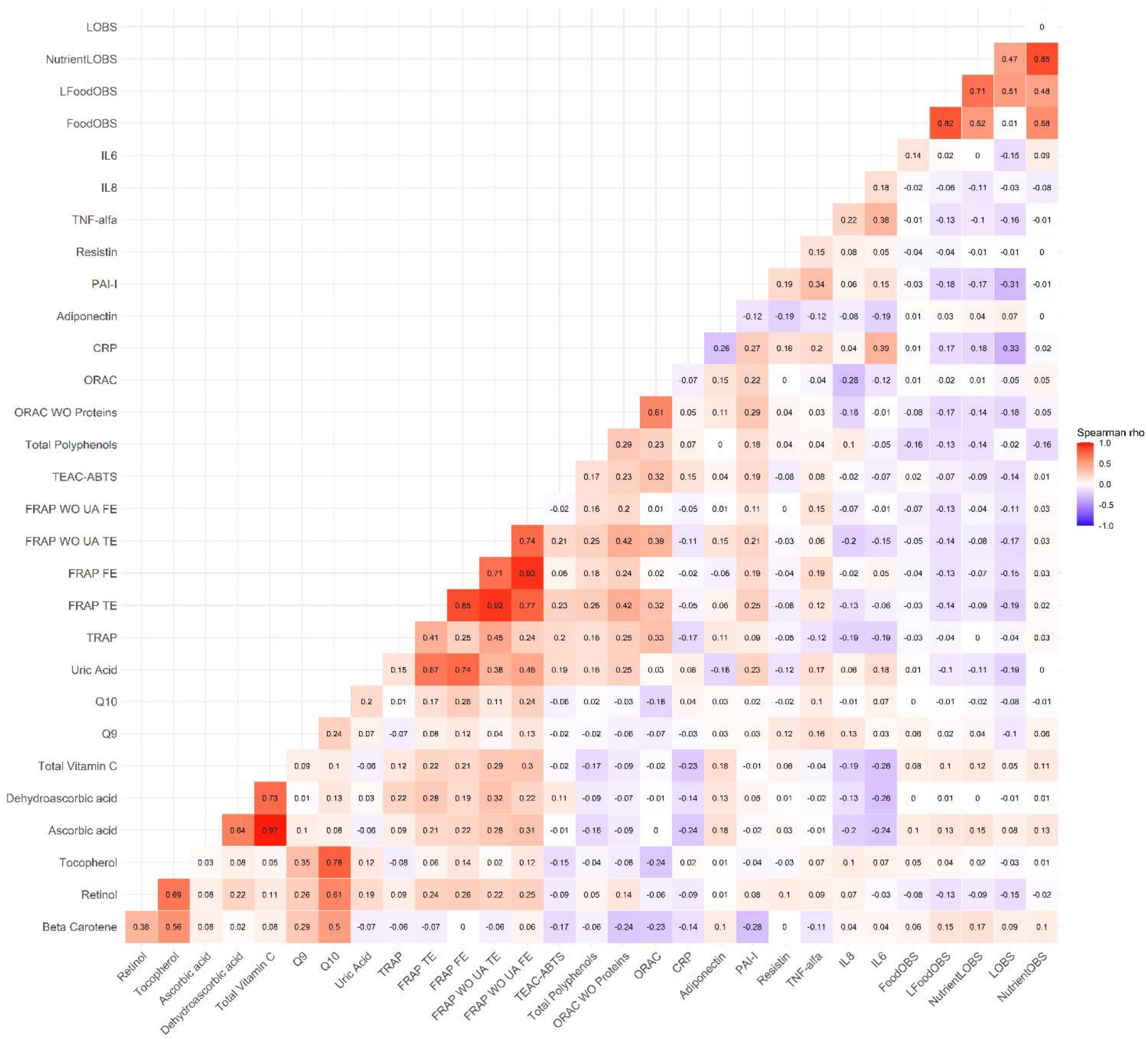

Supplementary Figure S1. Correlation matrix between the Nutrient, Lifestyle and Food Oxidative Balance Score (Nutrient-OBS, Nutrient-OBS, L-OBS, FoodL-OBS and Food-OBS) and the biomarkers of nutrient antioxidants (imputed data), OS and inflammation, in the EPIC Grana-da-Gipuzkoa subsample (N = 210).

The color value of the cells is proportional to the strength of the associations, ranging from red (positive correlations) to blue (negative correlations), as indicated in the color scale (at the right of the panel). Pair-wise spearman correlation coefficients (rho) are shown in every cell. Correlations above 0.3 were all statistically significant (p<0.05). Abbreviations: WO = without; UA = uric acid; OS = oxidative stress; CRP: C-reactive protein; PAI-I: Plasminogen activator; TNF-α: tumor necrosis factor; IL=Interleukin; TRAP: total radical-trapping antioxidant parameter; FRAP: ferric-reducing antioxidant power; TEAC-ABTS: trolox equivalent antioxidant capacity—Azino Bis Thiazoline Sulfonic; ORAC: oxygen radical absorbance capacity; TE: Trolox equivalents; FE: iron equivalents.

Biomarker missing values were imputed as described in the methods section
